# The Impact of SARS-CoV-2 Lineages (Variants) and COVID-19 Vaccination on the COVID-19 Epidemic in South Africa: Regression Study

**DOI:** 10.2196/34598

**Published:** 2023-07-03

**Authors:** Thabo Mabuka, Natalie Naidoo, Nesisa Ncube, Thabo Yiga, Michael Ross, Kuzivakwashe Kurehwa, Mothabisi Nare Nyathi, Andrea Silaji, Tinashe Ndemera, Tlaleng Lemeke, Ridwan Taiwo, Willie Macharia, Mthokozisi Sithole

**Affiliations:** 1 The Afrikan Research Initiative Motloung South Africa

**Keywords:** COVID-19, SARS-CoV-2, vaccines, variants, lineages, South Africa, epidemiology, statistics

## Abstract

**Background:**

Emerging SARS-CoV-2 variants have been attributed to the occurrence of secondary, tertiary, quaternary, and quinary COVID-19 epidemic waves threatening vaccine efforts owing to their immune invasiveness. Since the importation of SARS-CoV-2 in South Africa, with the first reported COVID-19 case on March 5, 2020, South Africa has observed 5 consecutive COVID-19 epidemic waves. The evolution of SARS-CoV-2 has played a major role in the resurgence of COVID-19 epidemic waves in South Africa and across the globe.

**Objective:**

We aimed to conduct descriptive and inferential statistical analysis on South African COVID-19 epidemiological data to investigate the impact of SARS-CoV-2 lineages and COVID-19 vaccinations in South African COVID-19 epidemiology.

**Methods:**

The general methodology involved the collation and stratification, covariance, regression analysis, normalization, and comparative inferential statistical analysis through null hypothesis testing (paired 2-tailed *t* tests) of South African COVID-19 epidemiological data.

**Results:**

The mean daily positive COVID-19 tests in South Africa’s first, second, third, fourth, and fifth COVID-19 epidemic wave periods were 11.5% (SD 8.58%), 11.5% (SD 8.45%), 13.3% (SD 9.72%), 13.1% (SD 9.91%), and 14.3% (SD 8.49%), respectively. The COVID-19 transmission rate in the first and second COVID-19 epidemic waves in South Africa was similar, while the COVID-19 transmission rate was higher in the third, fourth, and fifth COVID-19 epidemic waves than in the aforementioned waves. Most COVID-19 hospitalized cases in South Africa were in the general ward (60%-79.1%). Patients with COVID-19 on oxygen were the second-largest admission status (11.2%-16.8%), followed by patients with COVID-19 in the intensive care unit (8.07%-16.7%). Most patients hospitalized owing to COVID-19 in South Africa’s first, second, third, and fourth COVID-19 epidemic waves were aged between 40 and 49 years (16.8%-20.4%) and 50 and 59 years (19.8%-25.3%). Patients admitted to the hospital owing to COVID-19 in the age groups of 0 to 19 years were relatively low (1.98%-4.59%). In general, COVID-19 hospital admissions in South Africa for the age groups between 0 and 29 years increased after each consecutive COVID-19 epidemic wave, while for age groups between 30 and 79 years, hospital admissions decreased. Most COVID-19 hospitalization deaths in South Africa in the first, second, third, fourth, and fifth COVID-19 epidemic waves were in the ages of 50 to 59 years (15.8%-24.8%), 60 to 69 years (15.9%-29.5%), and 70 to 79 years (16.6%-20.7%).

**Conclusions:**

The relaxation of COVID-19 nonpharmaceutical intervention health policies in South Africa and the evolution of SARS-CoV-2 were associated with increased COVID-19 transmission and severity in the South African population. COVID-19 vaccination in South Africa was strongly associated with a decrease in COVID-19 hospitalization and severity in South Africa.

## Introduction

### Background

On March 11, 2020, the World Health Organization (WHO) declared COVID-19 a global pandemic [1]. The COVID-19 pandemic has resulted in >6,490,817 deaths in the reporting period until August 30, 2022 [2]. Public health measures, such as nationwide lockdowns aimed at reducing the transmission of COVID-19, have come at a great cost to the global economy [3]. There is a global consensus on the health risk posed by COVID-19, groundbreaking vaccine developments, and a great drive toward vaccination of the world population against COVID-19. However, challenges persist in controlling the global COVID-19 transmission and severity. One challenge is the large disparity in access to vaccines between low-income and high-income countries [4]. Another challenge is the emergence of SARS-CoV-2 lineages and sublineages (variants) with increased transmissibility [5]. Lineages and sublineages are a series of entities (in this case, genetic) forming a single line of direct ancestry and descent [6]. Emerging SARS-CoV-2 variants have been attributed to the occurrence of secondary, tertiary, quaternary, and quinary COVID-19 epidemic waves and threatening vaccine efforts owing to their immune invasiveness [7].

### SARS-CoV-2 Variants of Concern and Interest

SARS-CoV-2 is the virus that causes COVID-19 upon infecting a human host. Whole-genome sequencing of 104 strains of SARS-CoV-2 from patients with COVID-19 symptom onset from December 2019 to mid-February 2020 showed 99.9% homology, without major mutations [8]. However, the rapid spread of SARS-CoV-2 has allowed the virus opportune replications to evolve into lineages and sublineages. To prioritize global monitoring and research and to inform the ongoing response to the COVID-19 pandemic, SARS-CoV-2 variants have been characterized as either *variants of concern* (VOCs) or *variants of interest* (VOIs). The main characteristics of VOCs are that they have evidence of an increase in transmissibility and more severe disease that leads to increased hospitalization or deaths, thereby reducing the effectiveness of public health and social measures [9]. In addition, VOCs substantially reduce the neutralization of antibodies generated during previous infection or vaccination which ultimately reduces the effectiveness of treatments, vaccines, or diagnostic detection [10]. VOIs are lineages whose changes have predicted genetic markers that are known to affect virus characteristics, such as transmissibility, disease severity, immune escape, and diagnostic or therapeutic escape [9]. They are also identified to have a predictable increase in transmissibility or disease severity, thus having an apparent epidemiological impact to suggest an emerging risk to global public health [9,10]. The SARS-CoV-2 lineages that have been characterized as VOCs by the WHO are the alpha (B.1.1.7), beta (B.1.351, B1.351.2, and B.1.351.3), gamma (P.1, P.1.1, P.1.2, P.1.4, P1.6, and P.1.7), delta (B.1.617.2, AY.1, AY.2, AY.3, and AY.3.1), and omicron (B.1.1.529, BA.4, BA.5, BA.2.12.1, and BA.2.75) SARS-CoV-2 lineages [9,10]. The variants that have been characterized as VOIs are eta (B.1.525), iota (B.1.526), kappa (B.1.617.1), lambda (C.37), epsilon (B.1.427, B.1.429), zeta (P.2), theta (P.3), and mu (B.1.621) SARS-CoV-2 lineages [9].

### SARS-CoV-2 Lineages in South Africa

Of interest in this study is the impact of the evolution of SARS-CoV-2 lineages and COVID-19 vaccination in the COVID-19 epidemiology in South Africa. Since the importation of SARS-CoV-2 in South Africa, with the first reported COVID-19 case on March 5, 2020, South Africa has observed 5 consecutive COVID-19 epidemic waves [2,11]. The response by the Government of South Africa toward the COVID-19 epidemic was the establishment of a National Coronavirus Command Council to oversee the epidemic; the use of health policy measures, including nonpharmaceutical interventions (NPIs) to try to mitigate the transmission of COVID-19; and the implementation of COVID-19 vaccination programs to try to vaccinate the South African population against COVID-19 [12-17].

“Globally, systems have been established and are being strengthened to detect ‘signals’ of potential VOIs or VOCs and assess these based on the risk posed to global public health” [9]. In South Africa, the Network for Genomics Surveillance in South Africa was formed to understand the spread of SARS-CoV-2 [18]. During the first COVID-19 epidemic wave in South Africa, 16 SARS-CoV-2 lineages specific to South Africa were identified from 1365 high-quality whole genomes [18]. From these 16 lineages, 3 main clusters (B.1.1.54, B.1.1.56, and C.1 SARS-CoV-2 lineages) were identified to have caused approximately 42% of SARS-CoV-2 infections in South Africa [18]. Another sublineage specific to South Africa was the B.1.106 lineage that emerged in Kwa-Zulu Natal province in a nosocomial outbreak during the first COVID-19 epidemic wave [18]. The prevalence of this sublineage decreased as a result of control measures [18,19]. The C.1 lineage (first identified C lineage of SARS-CoV-2) was the most geographically spread lineage during the first COVID-19 epidemic wave in South Africa [18]. Before the resurgence of the second COVID-19 epidemic wave in South Africa, the beta (B.1.351, B1.351.2, and B.1.351.3 lineages) SARS-CoV-2 VOC (formerly GR/501Y.V2) was identified in an analysis of 2704 South African SARS-CoV-2 genotypes (samples collected till December 14, 2020) from the GISAID database. The beta (B.1.351 lineage) SARS-CoV-2 VOC was detected in samples collected in October 2020 [20]. The beta SARS-CoV-2 lineage became the dominant lineage in South Africa’s second COVID-19 epidemic wave, rapidly replacing the 3 main clusters (B.1.1.54, B.1.1.56, and C.1 SARS-CoV-2 lineages) identified during the first COVID-19 epidemic wave [20]. During the resurgence of the third COVID-19 epidemic wave in South Africa, 4 SARS-CoV-2 variants were identified: alpha, beta, eta, and delta SARS-CoV-2 variants. Genomic data for South African samples identified 65% of 1147 whole genomes from May 2021 as the beta SARS-CoV-2 variant. The alpha, delta, and eta SARS-CoV-2 variants accounted for 6%, 16%, and 1% of those samples, respectively. In June 2021, with 2931 genetic sequences in that period, the delta SARS-CoV-2 variant had become the dominant variant in samples collected in South Africa at 66%, while the beta and alpha SARS-CoV-2 variants accounted for 16% and 4%, respectively [21]. By the end of South Africa’s third COVID-19 epidemic wave in September 2021, the delta SARS-CoV-2 variant accounted for 96% of the 186 whole-genome sampled in that period, while the C1.2 SARS-CoV-2 lineage accounted for 1% of those samples [21]. The C1.2 SARS-CoV-2 lineage, a new South Africa-specific SARS-CoV-2 lineage (evolved from the C.1 SARS-CoV-2 lineage), was identified in South African samples in May 2021. The C.1.2 lineage was detected across the majority of South African provinces and in 7 other countries [22]. On November 25, 2021, the National Institute for Communicable Diseases (NICD) in South Africa confirmed the detection of the omicron SARS-CoV-2 VOC (B.1.1.529 lineage) in SARS-CoV-2 genomes of 22 laboratory-confirmed cases of COVID-19 [23]. The investigation into the initially identified cases of the B.1.1.529 SARS-CoV-2 lineage in South Africa was triggered by the absence of the S gene (S-gene dropout or-gene target failure) in a specific PCR assay because of the 69-70del deletion [24]. A similar observation was made during the early identification of the alpha SARS-CoV-2 VOC (B.1.1.7 lineage). The omicron SARS-CoV-2 VOC (B.1.1.529 lineage) was the dominant SARS-CoV-2 lineage in the fourth COVID-19 wave in South Africa, accounting for 82% of SARS-CoV-2 infections in November 2021 and 98.5% of SARS-CoV-2 infections in South Africa by January and February 2022 [25]. By the time of the resurgence of the fifth COVID-19 epidemic wave in South Africa in April 2022, the omicron SARS-CoV-2 VOC had evolved into sublineages. A total of 2459 whole genomes from South African samples with confirmed SARS-CoV-2 infection in April 2022 identified the omicron SARS-CoV-2 sublineages (BA.4, BA.2, and BA.5) in 54%, 25%, and 19% of the respective samples, respectively [26]. By the end of the fifth COVID-19 epidemic wave in South Africa in July 2022, 339 whole genomes from South African samples with confirmed SARS-CoV-2 infection in that period were identified to be largely omicron SARS-CoV-2 sublineages (BA.5 and BA.4), accounting for 96% of those samples [27].

### COVID-19 Vaccination in South Africa

The immunity against COVID-19 in humans is thought to be both innate and adaptive. Most patients with COVID-19 who recovered developed antibodies against SARS-CoV-2 within 1 to 3 weeks [28]. The SARS-COV-2 seroprevalence in South Africa’s first COVID-19 epidemic wave was estimated to be between 31% and 46% [29], while in the second COVID-19 epidemic wave, it was estimated to be 35.8% [30-32]. COVID-19 vaccination in South Africa commenced during South Africa’s second COVID-19 epidemic wave and was limited to frontline workers, such as health care workers, owing to the limited access to vaccines at the time [33]. COVID-19 vaccines were then largely administered to the rest of the South African population during the third and fourth COVID-19 epidemic waves. According to the WHO, there are currently 52 COVID-19 candidate vaccines in the clinical evaluation stages, of which 13 are in phase 3. Some vaccines have been reported to have an efficacy greater than 90% (BioNTech or Fosun Pharma or Pfizer, AstraZeneca, Sinovac, and Sputnik V vaccines) [28]. South Africa’s COVID-19 vaccination run through COVID-19 vaccination programs and clinical trials have largely administered the Pfizer-BioNTech (Comirnaty) and the Johnson & Johnson or Janssen doses to the South African population. By August 26, 2022, a total of 37.4 million people had been vaccinated against COVID-19 in South Africa, mainly with the Pfizer-BioNTech (Comirnaty) and the Johnson & Johnson or Janssen COVID-19 vaccines [34]. The Johnson & Johnson or Janssen COVID-19 vaccine is a viral vector vaccine, whereas the Pfizer-BioNTech (Comirnaty) COVID-19 vaccine is an mRNA vaccine. Both vaccines are dependent on the encoded SARS-CoV-2 spike (S) proteins to induce an immune response after vaccination. Thus, SARS-CoV-2 VOCs and VOIs with mutations on the spike (S) proteins emerging after the development of these vaccines have hampered their efficacy, requiring booster doses [35].

The evolution of SARS-CoV-2 has played a major role in the resurgence of the COVID-19 epidemic waves in South Africa and across the globe. South Africa has a unique observation of the evolution of SARS-CoV-2, with distinct SARS-CoV-2 lineages dominating certain epidemic periods. This unique observation allows for an investigation of the impact of the detected SARS-CoV-2 lineages on COVID-19 transmissibility and severity through the analysis of epidemiological data. In this study, a descriptive and inferential statistical analysis was conducted on South African COVID-19 epidemiological data to describe and investigate the impact of SARS-CoV-2 lineages and COVID-19 vaccinations on COVID-19 transmission and severity in the South African population.

## Methods

The general methodology in this study involved the collation and stratification of South African COVID-19 epidemiological data, covariance, regression analysis of epidemiological data, normalization, and comparative inferential statistical analysis through null hypothesis testing (paired 2-tailed *t* tests).

### Collation of South African COVID-19 Epidemiological Data

South African COVID-19 reported case data (cumulative and daily COVID-19 cases, recovered, and deaths) for the reporting period from January 22, 2020, to August 18, 2022, were obtained from the Johns Hopkins University Center for Systems Science and Engineering COVID-19 Database [2]. The South African COVID-19 testing data (cumulative and daily COVID-19 tests) were obtained from the Our World In Data project [36] for the reporting period from February 14, 2020, to June 22, 2022. South African COVID-19 hospitalization data were obtained from the NICD DATCOV surveillance system [37] for the period May 24, 2020, to August 18, 2022. The NICD DATCOV surveillance system in South Africa only started publishing reports on the reporting date of May 24, 2020; thus, data from March 5, 2020, to May 23, 2020, in the first COVID-19 epidemic wave period are missing. Data from October 9, 2020, to October 26, 2020, in the second COVID-19 epidemic wave period were also missing. The number of hospitals reporting to the NICD DATCOV surveillance system during South Africa’s first COVID-19 epidemic wave period was initially 204 facilities and the number of facilities increased to 666 by the end of the fifth COVID-19 epidemic wave period. The South African COVID-19 hospitalization data obtained in this study were composed of the number of facilities reporting, admission status data (daily COVID-19 hospital admission cases, hospitalized in high care, intensive care unit [ICU], isolation ward, on oxygen, and ventilator), cumulative COVID-19 admission age profile, cumulative COVID-19 hospital deaths age profile, and cumulative COVID-19 patients discharged alive. The COVID-19 hospital daily discharge rate (DR) and case fatality rate (CFR) were then calculated based on the methodology described in the study by Mabuka et al [38]. Weekly South African natural and excess (natural) deaths were obtained from the South African Medical Research Council [39] for the reporting period of December 29, 2019, to August 20, 2022. The weekly unreported excess deaths (natural) to COVID-19 death ratio (ECDR) was then calculated based on the methodology in the study by Mabuka et al [38].

South African COVID-19 vaccination data (cumulative number of people who received at least 1 dose of a COVID-19 vaccine per population age group) for the reporting period of February 17, 2021, to August 18, 2022, were obtained from the Department of Health Republic of South Africa COVID-19 Public Dashboard [34]. The data in the respective dashboards only contained COVID-19 vaccination records captured on the live Electronic Vaccination Data System and excluded vaccination records captured on paper [34]. South African community mobility data, which includes the change from baseline in movement in retail and recreation, grocery and pharmacy, parks, transit stations, workplaces, and residential locations, were obtained from the Google Community Mobility Reports [40] with data from the period of February 15, 2020, to August 18, 2022. The reports are created using aggregated data from users who have turned on their location history in their Google accounts. The baseline in these reports was the median values of movement in the respective locations from January 3 to February 6, 2020.

### Stratification of South African COVID-19 Epidemiological Data

Study analysis data sets from the collated South African COVID-19 epidemiological data were produced using SAS Base 9.4 software. To draw inferential comparisons regarding the impact of the evolution of SARS-CoV-2 and COVID-19 vaccinations in South African COVID-19 epidemiology, the collated South African COVID-19 epidemiological data were stratified based on the observed COVID-19 epidemic wave periods 1, 2, 3, 4, and 5 in South Africa. The South African COVID-19 epidemic wave periods 1, 2, 3, 4, and 5 were classified as collated data from March 5 to September 30, 2020; October 1, 2020, to April 26, 2021; April 27 to November 14, 2021; November 15, 2021, to April 1, 2022; and April 2 to July 31, 2022, respectively. The labels of stratified variables were given a suffix reference of “1,” “2,” “3,” “4,” and “5” for the 5 COVID-19 epidemic periods, respectively. For cumulative epidemiological data (South African cumulative COVID-19 admission age profile, cumulative COVIID-19 hospital deaths age profile, and cumulative COVID-19 patients discharged alive), the data were adjusted using equation 1 to remove the cumulative data from the previous COVID-19 epidemic period:

Cumulative epidemic variable adj (n), i = cumulative epidemic variable (n), i − cumulative epidemic variable (n), j **(1)**

where n is the number of patients, i is the reported date, and j is the last reported date of the previous COVID-19 epidemic period. The stratification of data in this study was done by splitting the data using the epidemic period variable in SPSS (version 28; IBM Corp).

On the basis of a review of the literature [18,20,21,25-27,41], Table S1 in Multimedia Appendix 1 summarizes the South African SARS-CoV-2 lineage clusters observed in the South African genomic data during the respective observed COVID-19 epidemic wave periods. On the basis of this stratification, the cluster of lineages identified in Table S1 in Multimedia Appendix 1 was assumed to be the SARS-CoV-2 lineages resulting in the respective COVID-19 epidemic waves in South Africa.

### Covariance and Regression of South African Epidemiological Data

In this study, covariance between the following epidemiological data was investigated: (1) COVID-19 daily tests and cases, (2) the number of reporting hospitals to NICD DATCOV and COVID-19 hospitalized cases, (3) COVID-19 active and hospitalized cases, (4) daily COVID-19 cases, change in daily COVID-19 cases, hospital-to-active cases (HA), CFR, DR, admission status, and admitted death status with daily cumulative COVID-19 vaccinated people. The covariance was investigated by applying Bivariate Analysis using 2-tailed Pearson and Spearman tests in SPSS. The 2 respective parametric and nonparametric methods were applied, considering the distribution of data and linearity. The assumed linearity was based on 2-tailed Pearson correlations with Pearson correlations above 0.850 or below 0.850, with *P<*.001 considered as having a high degree of linearity. For data with assumed linearity, further analysis was conducted by applying an ANOVA using the Univariate General Linear Model.

### Normalization and Paired *t* tests on South African Epidemiological Data

In this study, COVID-19 transmissibility was measured through the magnitude of mean and variance of the percentage of daily COVID-19 positive tests, considering the linear positive correlation between daily COVID-19 tests and cases. COVID-19 severity was measured through the magnitude of the mean and variance of the COVID-19 hospital admission cases, admission status, admission age profile, death age profile, CFR, DR, and ECDR. Considering the linear positive correlation between COVID-19 active and hospital admission cases, a normalized parameter (COVID-19 HA) was used to normalize the variance of active cases in hospital admission cases.

For the comparative inferential statistical analysis conducted to understand the impact of SARS-CoV-2 evolution on COVID-19 transmissibility and severity in South Africa, descriptive statistics and paired samples *t* test at 95% CIs were conducted on the normalized parameters in SPSS. The paired *t* test was conducted between the COVID-19 epidemic wave periods based on the following *t* test pairings: normalized parameter 1 with normalized parameter 2 (pair 1), normalized parameter 1 with normalized parameter 3 (pair 2), normalized parameter 1 with normalized parameter 4 (pair 3), normalized parameter 1 with normalized parameter 5 (pair 4), normalized parameter 2 with normalized parameter 3 (pair 5), normalized parameter 3 with normalized parameter 4 (pair 6), and normalized parameter 4 with normalized parameter 5 (pair 7) were the suffix number representing the COVID-19 epidemic wave period.

### Data Handling and Limitations

The propagated error due to data capturing or data reliability from data sources were not accounted for. Each descriptive and inferential statistical analysis test was applied independently to the analysis data sets, and there was no codependent or propagated error in the results of the applied statistical tests. The time variance of data was accounted for by reporting values to 95% CIs. Missing data values were not included in the analysis. The Pearson and Spearman correlation coefficients are limited to determining the strength of the association between ≥2 or more variables; however, they do not determine the causality of this relationship. In this study, COVID-19 seroprevalence was not investigated as a confounder because of the limitations of these data.

### Ethics Approval

An internal ethical assessment was conducted within the Afrikan Research Initiative at the start of the ARI COVID-19 Research Project, and no regional ethics approval was requested for this study. Data used in this study were obtained from public sources with an Open Data Licence. Patient data were obtained from the public source and anonymized following the local regulations of the Protection of Personal Information Act in South Africa.

## Results

### Covariance and Regression of South African COVID-19 Epidemiological Data

Table 1 shows that the mean COVID-19 daily tests in the first, second, third, fourth, and fifth South African COVID-19 epidemic wave periods were 20,575 (SD 14,062), 31,046 (SD 14,115), 41,315 (SD 16,108), 35,226 (SD 17,078), and 23,419 (SD 7229), respectively.

**Table 1:** Statistical sample number (n), range, mean (SD), and coefficient of skewness of daily COVID-19 tests and cases in the first, second, third, fourth, and fifth COVID-19 epidemic wave periods in South Africa (descriptive statistics).

Parameter and COVID-19 epidemic wave		Values, n^a^	Values, mean (SD; range)	Skewness
**Daily COVID-19 cases**				
	1	237	2845 (3794; 0-13,944)	1.53
	2	208	4336 (5034; 437-21,980)	1.79
	3	197	6672 (6252; 0-26,485)	0.84
	4	137	5705 (6816; 0-37,875)	1.95
	5	82	3293 (2791; 0-13,613)	1.31
**Daily COVID-19 tests**				
	1	203	20,575 (14,062; 4-56,663)	0.47
	2	201	31,046 (14,115; 10,402-77,167)	1.16
	3	191	41,315 (16,108; 13,507-96,896)	0.62
	4	133	35,226 (17,078; 12,942-104,831)	1.40
	5	61	23,419 (7299; 9149-39,613)	0.12

^a^Values, n represents the number of observations or records pooled for the mean sample size calculations. All other values represent absolute descriptive statistical values.

The Pearson (coefficient of determination) correlation coefficients between COVID-19 daily tests (independent variable) and cases (dependent variable) in the first, second, third, fourth, and fifth COVID-19 epidemic waves in South Africa were determined to be 0.910 (0.828), 0.877 (0.769), 0.893 (0.797), 0.859 (0.737), and 0.749 (0.562), respectively (shown in Table S2 in Multimedia Appendix 1). The *F* test values between the mean square regression and residual for the daily COVID-19 tests and cases linear regression in the first, second, third, fourth, and fifth COVID-19 epidemic waves in South Africa are shown in Table S3 in Multimedia Appendix 1, and the unstandardized and standardized coefficients are shown in Table S4 in Multimedia Appendix 1. The Pearson (coefficient of determination) correlation coefficients between facilities reporting to the NICD DATCoV (independent variable) and COVID-19 hospital admission cases (dependent variable) in the first, second, third, and fourth COVID-19 epidemic waves in South Africa were 0.336 (0.113), 0.212 (0.045), 0.385 (0.148), and −0.249 (−0.062), respectively (Table S5 in Multimedia Appendix 1). The Pearson (coefficient of determination) correlation coefficients between COVID-19 active (independent variable) and hospital admission cases (dependent variable) in the first, second, third, fourth, and fifth COVID-19 epidemic waves in South Africa were 0.932 (0.869), 0.819 (0.671), 0.967 (0.936), 0.919 (0.845), and 0.863 (0.745), respectively (Table S5 in Multimedia Appendix 1). The unstandardized and standardized coefficients between the daily COVID-19 tests and cases in the first, second, third, fourth, and fifth COVID-19 epidemic waves in South Africa are presented in Table S6 in Multimedia Appendix 1.

South Africa’s COVID-19 NPI health policy response to the COVID-19 epidemic waves in South Africa was implemented in the form of National Lockdown Alert Level policies. The National Lockdown Alert Level policies were largely entry and exit screening at borders, limitations of movements and gatherings, closure and limitations of institutions and business activities, ban and limiting of alcohol and tobacco industries, isolation, quarantine of potentially infected persons, contact-tracing protocols, use of personal protective equipment, and hygienic protocols [38,42]. The adjustment in the alert levels resulted in eased movement restrictions compared with their predecessors. Table S7 in Multimedia Appendix 1 shows the summary of COVID-19 NPI policies implemented in South Africa during the first, second, third, fourth, and fifth COVID-19 epidemic waves.

**Figure 1:**
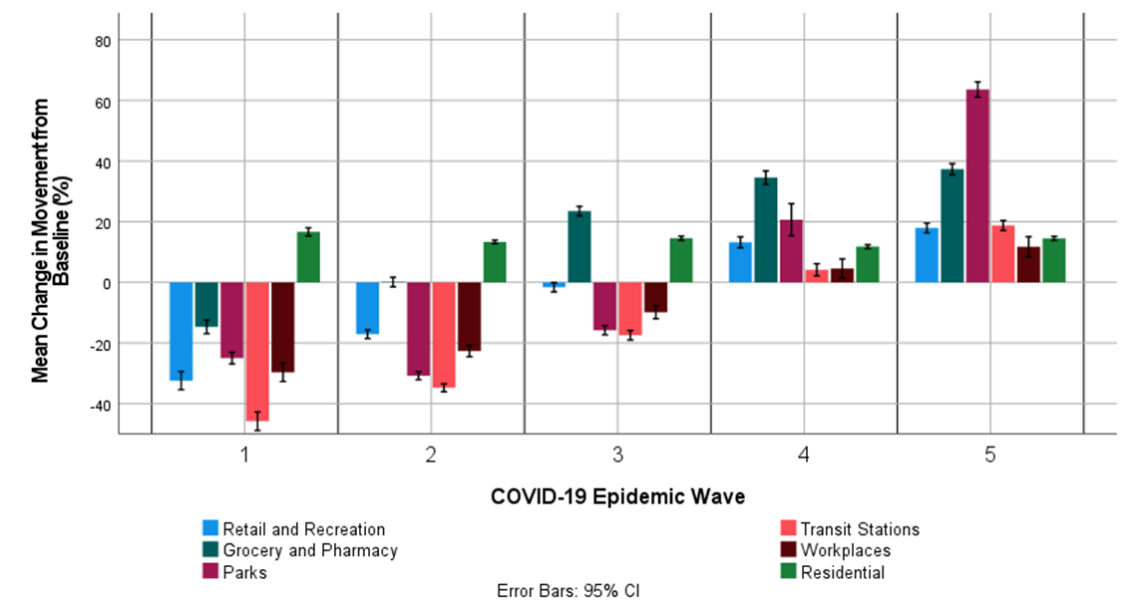
Mean change in movement from baseline in retail and recreation, grocery and pharmacy, parks, transit stations, workplaces, and residences during the South African first, second, third, fourth, and fifth COVID-19 epidemic waves.

The mean change in movement from baseline in the retail and recreation, grocery and pharmacy, parks, transit stations, workspaces, and residential locations in South Africa during the implementation of the no national lockdown; National Lockdown Level 5, 4, 3, and 2; and National Lockdown Adjusted Level 4, 3, 2, and 1 are shown in Multimedia Appendix 2. In general, COVID-19 NPI policies in South Africa resulted in a negative mean change in movement from baseline in retail and recreation, grocery and pharmacy, parks, transit stations, and workplaces. Residential locations in South Africa had a positive mean change in movement from baseline. A decrease in the national lockdown alert levels resulted in a decrease in the modulus mean change in movement from the baseline in locations in South Africa. The national lockdown adjusted alert levels resulted in a lower modulus mean change in movement from the baseline in locations in South Africa than the lowest unadjusted Alert Level (National Lockdown Alert Level 2). Concerning movement, the most affected locations in South Africa by the COVID-19 NPI policies implemented were the retail and recreation, transit stations, workplaces, and residential locations. The National Lockdown Alert Level 5 had the largest impact on the movement in South African communities, whereas the National Lockdown Adjusted Alert Level 1 had the least impact on movement in South African communities. Figure 1 shows the mean change in movement from baseline in retail and recreation, grocery and pharmacy, parks, transit stations, workspaces, and residential locations in South Africa during the first, second, third, fourth, and fifth COVID-19 epidemic waves in South Africa. Figure 1 shows that the negative mean change in movement from baseline in retail and recreation, grocery and pharmacy, parks, transit stations, and workspaces gradually decreased during each consecutive COVID-19 epidemic wave. The mean change in movement from the baseline in residential locations was positive for the 5 COVID-19 epidemic waves. Figure 1 also shows that the mean change in movement from baseline in South African locations was positive during the fourth and fifth COVID-19 epidemic waves.

**Figure 2:**
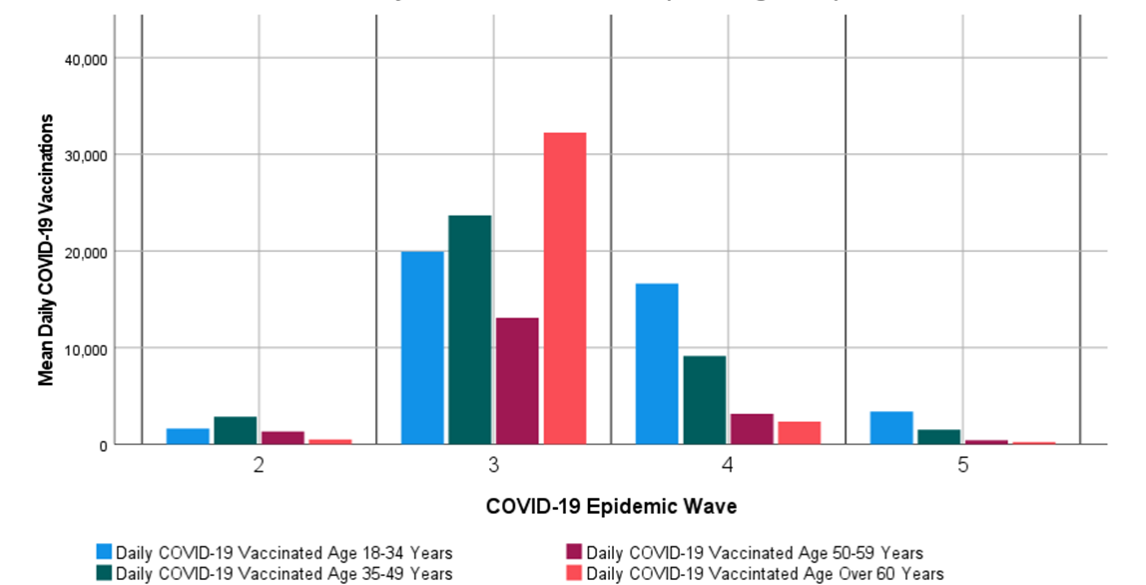
Mean daily number of COVID-19 vaccinated people per age group during the first, second, third, fourth, and fifth COVID-19 epidemic wave in South Africa.

The cumulative (maximum) number of people receiving at least 1 dose of the COVID-19 vaccine relative to the total population per age group in the first, second, third, fourth, and fifth COVID-19 epidemic waves is shown in Multimedia Appendix 3. By August 30, 2022, a total of 37,456,345 doses of COVID-19 vaccines had been administered in South Africa. Of the 37,456,345 nationally administered doses, 9,190,172 (24.5%) doses of the COVID-19 vaccine administered were the Johnson & Johnson COVID-19 vaccine, with 1,385,476 (3.7%) doses being the Johnson & Johnson COVID-19 vaccine booster dose; 28,266,173 (75.5%) of the national administered amount was the Pfizer COVID-19 vaccine, with 14,452,185 (38.6%), 11,515,875 (30.7%), and 2,298,113 (6.14%) being the Pfizer COVID-19 vaccine first, second, and third booster doses, respectively [34]. Figure 2 shows the mean daily number of COVID-19 vaccinated people per age group during the first, second, third, fourth, and fifth COVID-19 epidemic waves in South Africa. Figure 2 shows that the mean daily COVID-19 vaccination rate per age group was lowest in the second and fourth COVID-19 epidemic waves and highest in the third COVID-19 epidemic wave for all age groups. There were no reported administered COVID-19 vaccinations in South Africa during the first COVID-19 epidemic wave. Figure 2 shows that there was a decrease in the mean daily COVID-19 vaccination rate in all age groups after the third COVID-19 epidemic wave.

The Spearman correlation coefficients and *P* values among daily COVID-19 cases, change in daily COVID-19 cases, HA, CFR, DR, admission status, and admitted death status with daily cumulative COVID-19 vaccinated people are shown in Table S8 in Multimedia Appendix 1. The Spearman correlation coefficients and *P* values between the daily cumulative COVID-19 vaccinated people and the daily COVID-19 cases in the first half period of the third, fourth, and fifth COVID-19 epidemic waves in South Africa were 0.930 (95% CI 0.890-0.956), 0.842 (95% CI 0.713-0.916), and 0.811 (95% CI 0.673-0.895), respectively, with *P<*.001. The Spearman correlation coefficients and *P* values between the daily cumulative COVID-19 vaccinated people and the change in daily COVID-19 cases were 0.031 (*P=*.79; 95% CI −0.207 to 0.266), −0.014 (*P=*.93; 95% CI −0.341 to 0.316), and −0.077 (*P=*.62; 95% CI −0.374 to 0.233), respectively. These results show a significant strong positive monotonic correlation (*P<*.001) between daily COVID-19 cases and daily cumulative COVID-19 vaccinations and a weak monotonic correlation between the change in daily COVID-19 cases and daily cumulative COVID-19 vaccinations. The Spearman correlation coefficients and *P* values between the daily cumulative COVID-19 vaccinated people and the daily HA were −0.983 (95% CI −0.989 to −0.972), −0.852 (*P=*.93; 95% CI −0.921 to −0.731), and −0.917 (*P=*.62; 95% CI −0.955 to −0.850), respectively, with *P<*.001. These results show a significantly strong negative monotonic correlation (*P<*.001) between daily COVID-19 HA and daily cumulative COVID-19 vaccinated individuals. The Spearman correlation coefficients and *P* values between the daily cumulative COVID-19 vaccinated people and the CFR were 0.380 (*P<*.001; 95% CI 0.160-0.565), 0.192 (*P=*.25; 95% CI −0.150 to 0.494), and 0.264 (*P=*.09; 95% CI −0.049 to 0.529), respectively. These results show a moderate positive monotonic correlation between hospital CFR and daily cumulative COVID-19 vaccinated people. The Spearman correlation coefficients between daily cumulative COVID-19 vaccinated people and the percentage hospitalized age groups of 18 to 34 years showed a significantly strong positive monotonic correlation (*P<*.001), while the age groups of 50 to above 60 years showed a significantly strong negative monotonic correlation (*P<*.001).

### Descriptive Statistics and Paired Samples *t* tests of South African COVID-19 Epidemiological Data

#### COVID-19 Detection in South Africa

Table 2 shows the descriptive statistics for COVID-19 active and daily positive COVID-19 tests for the first, second, third, fourth, and fifth COVID-19 epidemic wave periods in South Africa.

**Figure 3:**
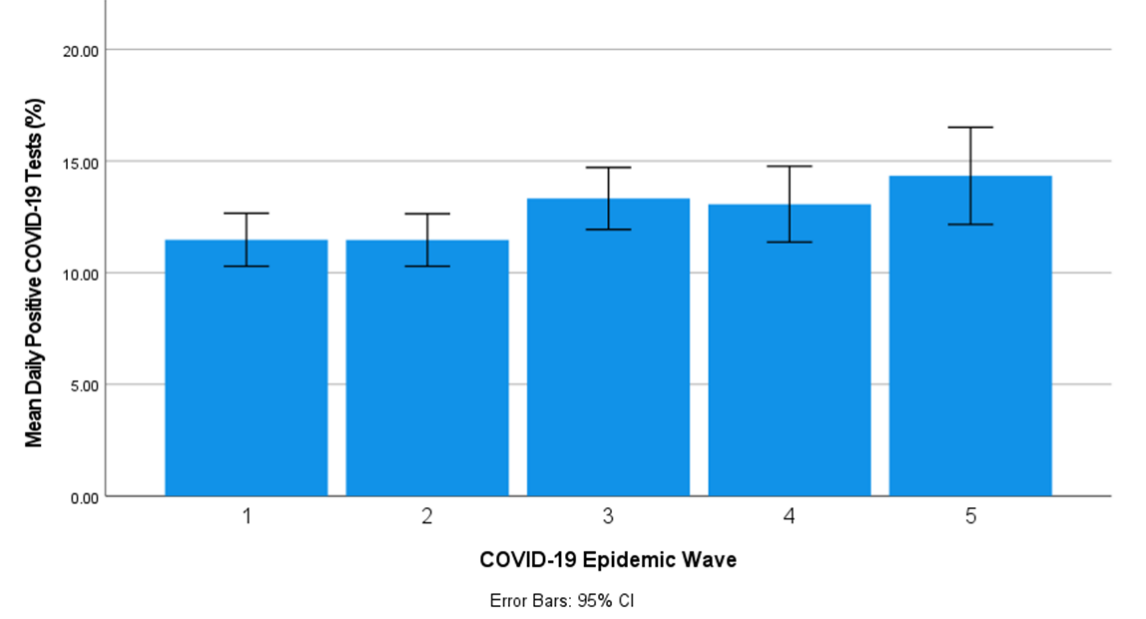
The mean daily positive COVID-19 tests (with 95% CI error bars) in the first, second, third, fourth, and fifth COVID-19 epidemic wave period in South Africa.

The daily positive COVID-19 tests indicate the transmissibility of COVID-19 based on the detection rate of COVID-19 and account for covariance in the testing rate for statistical comparison between the epidemic wave periods. The mean daily positive COVID-19 tests in South Africa’s first and second COVID-19 epidemic waves were not statistically different (*P*=.97). The mean daily positive COVID-19 tests in South Africa’s third, fourth, and fifth COVID-19 epidemic waves were 15.7%, 18.4%, and 24.3% more than those of the first and second COVID-19 epidemic wave periods, respectively. The difference among the mean daily positive COVID-19 tests in South Africa’s first, second, third, fourth, and fifth COVID-19 epidemic wave periods can also be observed in Figure 3. A paired *t* test of the daily positive COVID-19 tests between the first and second COVID-19 epidemic wave periods (pair 1) showed no significant difference at a 95% CI between these COVID-19 epidemic periods with **P*=*.97 (Table 3). The paired *t* test of the daily positive COVID-19 tests between test pairs 2 to pair 7 showed statistically significant differences at a 95% CI between the respective COVID-19 epidemic periods (Table 3).

**Table 2:** Statistical sample number (n), range, and mean (SD) of COVID-19 active cases and daily positive COVID-19 tests in the first, second, third, fourth, and fifth COVID-19 epidemic wave periods in South Africa (descriptive statistics).

Parameter and COVID-19 epidemic wave			Values, n^a^		Values, mean (SD; range)
**COVID-19 active cases**					
	1	237		45,851 (53,975; 0-173,590)	
	2	208		66,178 (53,878; 19,809-239,799)	
	3	197		89,171 (63,613; 16,243-211,052)	
	4	137		75,527 (65,996; 10,849-216,947)	
	5	82		35,833 (22,323; 9644-81,174)	
**Daily positive COVID-19 tests (%)**					
	1	203		11.5 (8.58; 0-34.0)	
	2	201		11.5 (8.45; 2.7-33.7)	
	3	191		13.3 (9.72; 0-31.6)	
	4	133		13.3 (9.91; 0-60.7)	
	5	61		13.3 (8.49; 0-41.6)	

^a^Values, n represents the number of observations or records pooled for the mean sample calculations. All other values represent absolute descriptive statistical values.

**Table 3:** Mean paired differences, SD of paired differences, SE of mean (SE mean), 95% CI of the upper and lower difference, t value, df, and *P* value (significance, 2-tailed) for the daily positive COVID-19 tests in pair 1 to pair 7 (paired samples *t* test).

Sample *t* test pairing	Paired variables	Paired differences, mean (SD)	*t* test (*df*)	Significance (2-tailed), *P* value
Pair 1	Daily positive COVID Test1—daily positive COVID Test2	−0.03 (13.26)	−0.04 (198)	.97
Pair 2	Daily positive COVID Test1—daily positive COVID Test3	−2.78 (14.42)	−2.65 (187)	.009
Pair 3	Daily positive COVID Test1—daily positive COVID Test4	−5.00 (15.10)	−3.66 (121)	*<*.001
Pair 4	Daily positive COVID Test1—daily positive COVID Test5	−10.8 (10.67)	−7.39 (52)	*<*.001
Pair 5	Daily positive COVID Test2—daily positive COVID Test3	−1.20 (5.95)	−2.80 (191)	.006
Pair 6	Daily positive COVID Test3—daily positive COVID Test4	5.22 (14.87)	3.96 (126)	*<*.001
Pair 7	Daily positive COVID Test4—daily positive COVID Test5	2.71 (8.85)	2.37 (59)	.02

#### COVID-19 Hospital Admissions in South Africa

**Figure 4:**
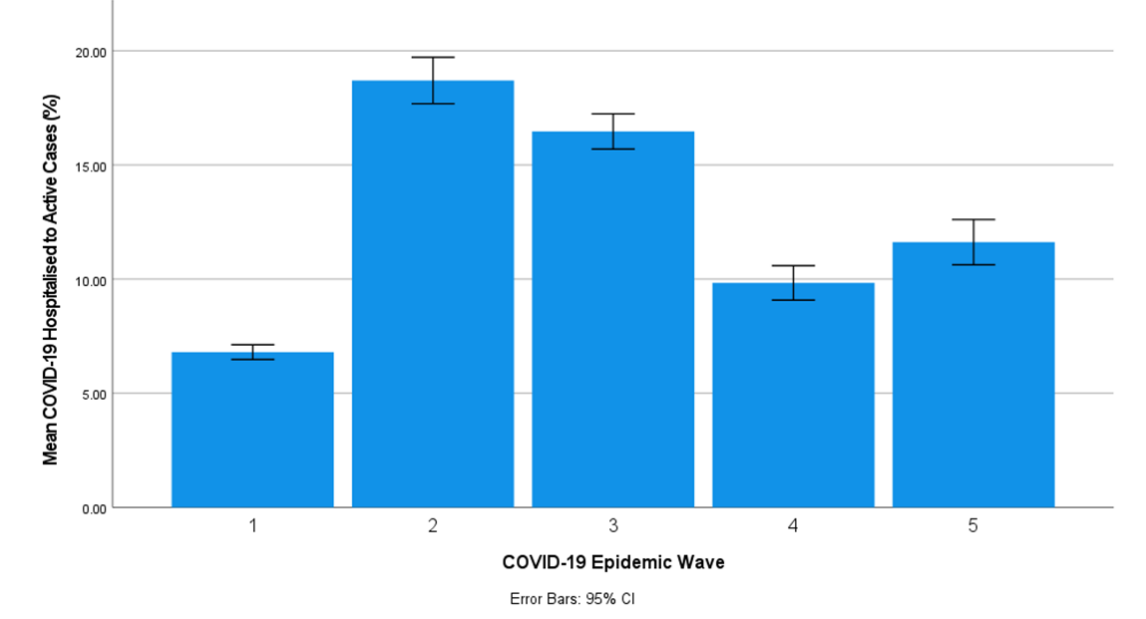
The mean COVID-19 hospitalized-to-active cases (with 95% CI error bars) in the first, second, third, fourth, and fifth COVID-19 epidemic wave period in South Africa.

Table 4 shows the descriptive statistics for the COVID-19 HA for the first, second, third, fourth, and fifth COVID-19 epidemic wave periods in South Africa. The second COVID-19 epidemic wave period in South Africa had the highest number of COVID-19 HA, followed by the third COVID-19 epidemic wave period. The mean paired difference of the COVID-19 HA in pairs 1, 2, 3, 4, 5, 6, and 7 was −14.0%, −10.6%, −4.62%, −7.72%, 2.31%, 4.02%, and −2.34%, respectively (Table 5). The difference between the mean number of COVID-19 HA in South Africa’s first, second, third, fourth, and fifth COVID-19 epidemic wave periods can also be observed in Figure 4. Paired *t* tests of the COVID-19 HA among pairs 1, 2, 3, 4, 5, 6, and 7 showed a significant difference at a 95% CI between the respective COVID-19 epidemic periods with *P<*.001 (Table 5).

Multimedia Appendix 4, Multimedia Appendix 5, and Multimedia Appendix 6 show the COVID-19 HA and vaccinated people in the first half period of the third, fourth, and fifth COVID-19 epidemic wave in South Africa, respectively. The first half period of the COVID-19 epidemic wave is the period from the first case to the peak of the epidemic wave. Multimedia Appendices 4-6 show that the number of COVID-19 HA decreased while the number of COVID-19 vaccinated people increased in the first half period of the third, fourth,^,^ and fifth COVID-19 epidemic wave in South Africa.

**Table 4:** Statistical sample number (N), minimum, maximum, and mean (SD) of COVID-19 hospital-to-active cases in the first, second, third, fourth, and fifth COVID-19 epidemic wave periods in South Africa (descriptive statistics).

Parameter and COVID-19 epidemic wave		Values, N^a^	Values, mean (SD; range)
**COVID-19 hospital-to-active cases (%)**			
	1	126	6.80 (1.82; 4.02-12.7)
	2	189	18.7 (7.08; 0-32.4)
	3	202	16.5 (5.56; 9.28-29.0)
	4	138	9.84 (4.48; 3.65-24.2)
	5	113	4.48 (5.32; 3.73-21.7)

^a^Values, n represents the number of observations or records pooled for the mean sample calculations. All other values represent absolute descriptive statistical values.

**Table 5:** Mean paired differences, SD of paired differences, SE of mean (SE mean), t value, df, and *P* value (significance, 2-tailed) for the COVID-19 hospital-to-active (HA) cases in pair 1 to pair 7 (paired samples *t* test).

Sample *t* test pairing	Paired variables	Paired differences, mean (SD)	*t* test (*df*)	Significance (2-tailed), *P* value
Pair 1	HA1—HA2	−14.0 (8.59)	−18.3 (126)	*<*.001
Pair 2	HA1—HA3	−10.6 (5.99)	−19.7 (122)	*<*.001
Pair 3	HA1—HA4	−4.62 (4.41)	−7.48 (50)	*<*.001
Pair 4	HA1—HA5	−7.72 (4.01)	−11.2 (33)	*<*.001
Pair 5	HA2—HA3	2.31 (7.34)	4.33 (189)	*<*.001
Pair 6	HA3—HA4	4.02 (5.21)	9.07 (137)	*<*.001
Pair 7	HA4—HA5	−2.34 (3.04)	−8.42 (119)	*<*.001

#### COVID-19 Hospital Admission Status in South Africa

**Figure 5:**
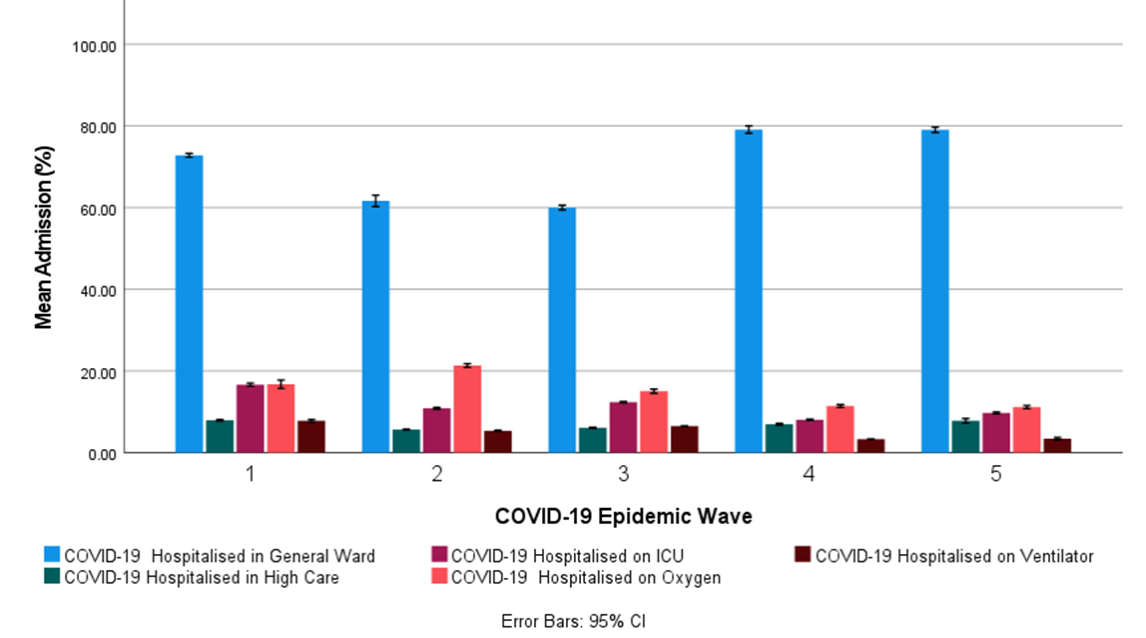
The mean COVID-19 hospitalized admission status (with 95% CI error bars) in the first, second, third, fourth, and fifth COVID-19 epidemic wave period in South Africa.

Table 6 shows the descriptive statistics for the COVID-19 hospital admission status for the first, second, third, fourth, and fifth COVID-19 epidemic wave periods in South Africa. Figure 5 shows the COVID-19 hospital admission status profiles in the first, second, third, fourth, and fifth COVID-19 epidemic wave periods in South Africa. Figure 5 shows that most COVID-19 hospitalized cases in South Africa were hospitalized in the general ward (60.0%-79.1%). Figure 5 also shows that the COVID-19 patients on oxygen were the second-largest admission status (11.2%-16.8%), followed by patients with COVID-19 in the ICU (8.07%-16.7%). The number of hospitalized COVID-19 cases admitted to the general ward was highest in the fourth and fifth COVID-19 epidemic wave periods, followed by the first COVID-19 epidemic wave period. The number of South African patients with COVID-19 admitted in high care, in the ICU, and on ventilators was highest in the first COVID-19 epidemic wave. The number of patients with COVID-19 on oxygen was highest in the second COVID-19 epidemic wave. The general trend showed a decrease in patients admitted on oxygen, in the ICU, and on a ventilator in the fourth and fifth COVID-19 epidemic waves compared with previous COVID-19 epidemic waves. Patients in high care have remained relatively in the mean range of 5.65% (SD 0.80%) to 7.93% (SD 1.03%) throughout the COVID-19 epidemic in South Africa. Paired *t* tests of the mean COVID-19 hospital admission status in pairs 1, 2, 3, 4, 5, 6, and 7 showed a significant difference at a 95% CI between the respective COVID-19 epidemic periods with *P<*.001 (Table S9 in Multimedia Appendix 1). Except for the COVID-19 hospitalized cases admitted in the general ward, in high care, on oxygen, and on ventilator between the fourth and fifth COVID-19 epidemic waves (pair 7), whose difference was found not to be statistically significant (Table S9 in Multimedia Appendix 1).

**Table 6:** Statistical sample number (n), range, and mean (SD) of COVID-19 hospitalized cases in the general ward, in high care, in the intensive care unit (ICU), on oxygen, and on ventilator in the first, second, third, fourth, and fifth COVID-19 epidemic wave periods in South Africa (descriptive statistics).

Parameter and COVID-19 epidemic wave			Values, n^a^		Values, mean (SD; range)		Variance
**COVID-19 hospitalized general ward (%)**							
	1	126		72.8 (2.62; 68.7-78.1)		6.9	
	2	187		61.7 (9.61; 52.6-86.2)		92.4	
	3	202		60 (4.10; 50.9-70.7)		16.8	
	4	138		79.1 (5.45; 63.8-84.4)		29.7	
	5	126		79.1 (3.71; 56.9-84.3)		13.8	
**COVID-19 hospitalized high care (%)**							
	1	126		7.93 (1.03; 5.07-10.5)		1.07	
	2	187		5.65 (0.80; 4.09-7.81)		0.647	
	3	202		6.09 (0.81; 4.51-7.80)		0.651	
	4	138		6.96 (1.33; 4.66-11.0)		1.76	
	5	126		7.81 (3.22; 4.29-30.0)		10.34	
**COVID-19 hospitalized ICU (%)**							
	1	126		16.7 (2.17; 11.9-20.6)		4.69	
	2	187		10.9 (1.49; 7.96-14.1)		2.21	
	3	202		12.4 (1.12; 9.65-15.1)		1.25	
	4	138		8.07 (0.89; 6.33-10.1)		0.789	
	5	126		9.74 (1.28; 6.97-11.8)		1.646	
**COVID-19 hospitalized on oxygen (%)**							
	1	126		16.8 (5.76; 10.6-30.8)		33.2	
	2	187		21.4 (3.12; 14.7-27.7)		9.72	
	3	202		15.1 (3.64; 8.43-23.9)		13.3	
	4	138		11.4 (2.02; 7.66-16.3)		4.07	
	5	126		11.2 (2.18; 8.81-26.3)		4.75	
**COVID-19 hospitalized on ventilator (%)**							
	1	126		7.81 (1.79; 4.87-10.8)		3.20	
	2	187		5.34 (1.17; 3.28-6.93)		1.38	
	3	202		6.49 (0.68; 3.86-8.08)		0.458	
	4	138		3.29 (0.45; 2.47-4.50)		0.205	
	5	126		3.41 (1.74; 2.33-16.3)		3.013	

^a^Values, n represents the number of observations or number of records pooled for the mean sample calculations. All other values represent the absolute descriptive statistical values.

#### COVID-19 Hospital Admission Age Profile in South Africa

Table 7 shows the descriptive statistics for the COVID-19 hospital admission age profiles for the first, second, third, fourth, and fifth COVID-19 epidemic wave periods in South Africa.

**Figure 6:**
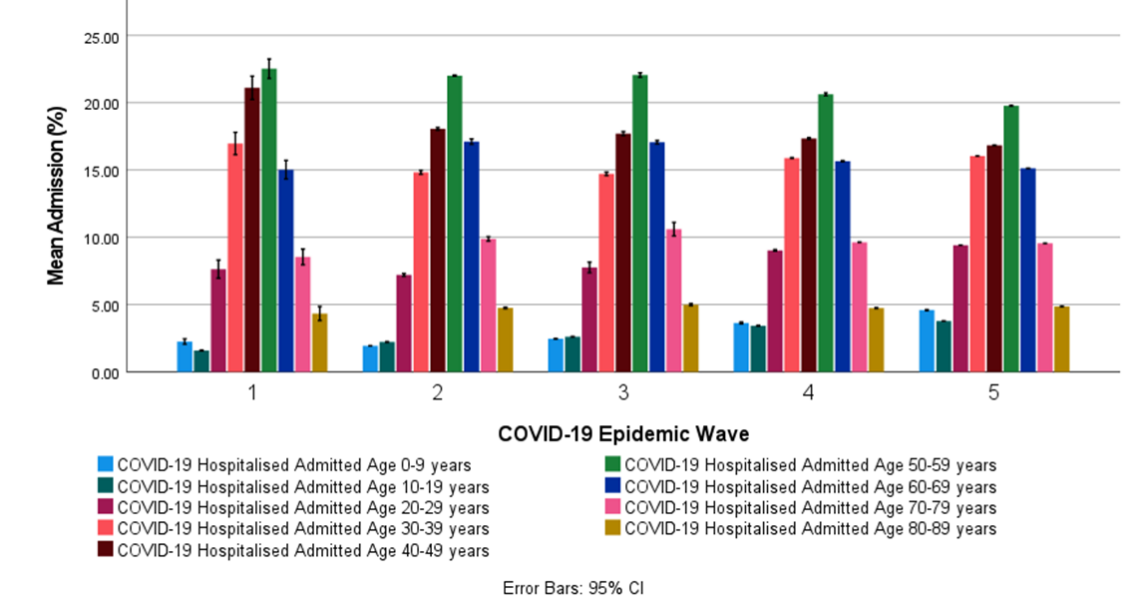
The mean COVID-19 hospitalized admission age profile (with 95% CI error bars) in the first, second, third, fourth, and fifth COVID-19 epidemic wave period in South Africa.

Figure 6 shows the COVID-19 hospital admission age profile in the first, second, third, fourth, and fifth COVID-19 epidemic wave periods in South Africa. Figure 6 shows that most COVID-19 hospitalized cases in South Africa’s first, second, third, and fourth COVID-19 epidemic wave periods were in the ages of 40 to 49 years (16.8%-20.4%) and 50 to 59 years (19.8%-25.3%), respectively. Patients admitted owing to COVID-19 in the age groups of 0 to 19 years were relatively low (1.98%-4.59%) and highest in the fourth and fifth COVID-19 epidemic wave periods. Figure 6 shows that the mean age profile for COVID-19 hospital admissions for the first, second, third, fourth, and fifth COVID-19 epidemic wave periods had a similar relative normal distribution within the admitted age groups. In general, COVID-19 hospital admissions in South Africa for the age groups between 0 and 29 years increased after each consecutive COVID-19 epidemic wave, while those for age groups between 30 and 79 years decreased.

Paired *t* tests of the COVID-19 hospital admission age profile between the COVID-19 epidemic waves for all age groups showed statistically significant differences at a 95% CI between the respective COVID-19 epidemic periods with *P<*.001 (Table S10 in Multimedia Appendix 1), except for the following:

- Age groups between 20 and 29 years, 50 and 59 and 60 and 69 years between COVID-19 epidemic waves 2 and 3 (*P*=.12, *P*=.08, and *P*=.68, respectively).

**Table 7:** Statistical sample number (n), range, mean, and SD of COVID-19 hospitalized cases in the ages of 0 to 9, 10 to 19, 20 to 29, 30 to 39, 40 to 49, 50 to 59, 60 to 69, 70 to 79, and 80 to 89 years in the first, second, third, fourth, and fifth COVID-19 epidemic wave periods in South Africa (descriptive statistics).

Parameter and COVID-19 epidemic wave			Values, n^a^		Values, mean (SD; range)		Variance
**COVID-19 hospitalized admitted (age 0-9 years; %)**							
	1	110		2.32 (0.58; 1.68-3.96)		0.34	
	2	187		1.98 (0.25; 1.81-3.66)		0.06	
	3	202		2.47 (0.19; 1.14-3.48)		0.03	
	4	138		3.63 (0.40; 2.92-4.14)		0.16	
	5	126		4.59 (0.21; 4.21-4.83)		0.05	
**COVID-19 hospitalized admitted (age 10-19 years; %)**							
	1	108		1.75 (0.22; 1.23-2.62)		0.05	
	2	179		2.23 (0.18; 1.98-2.56)		0.03	
	3	202		2.62 (0.20; 1.19-3.68)		0.04	
	4	138		3.43 (0.18; 3.09-3.66)		0.03	
	5	126		3.78 (0.03; 3.71-3.81)		0.00	
**COVID-19 hospitalized admitted (age 20-29 years; %)**							
	1	126		8.04 (2.11; 0.04-14.6)		4.46	
	2	187		7.39 (1.19; 0.63-13.8)		1.41	
	3	202		7.78 (2.83; 3.30-47.3)		8.02	
	4	138		9.03 (0.34; 8.29-9.32)		0.11	
	5	126		9.42 (0.01; 9.40-9.43)		0.00	
**COVID-19 hospitalized admitted (age 30-39 years; %)**							
	1	126		18.1 (3.27; 11.4-29.8)		10.68	
	2	187		15.3 (2.49; 13.0-30.5)		6.20	
	3	202		14.8 (0.98; 6.36-18.9)		0.96	
	4	138		15.9 (0.16; 15.5-16.0)		0.02	
	5	126		16.0 (0.05; 16.0-16.1)		0.00	
**COVID-19 hospitalized admitted (age 40-49 years; %)**							
	1	75		20.4 (2.64; 18.8-27.4)		6.99	
	2	181		18.17 (1.52; 17.2-36.5)		2.31	
	3	201		17.73 (1.11; 7.70-22.6)		1.23	
	4	138		17.3 (0.40; 17.0-18.6)		0.16	
	5	126		16.8 (0.13; 16.7-17.0)		0.02	
**COVID-19 hospitalized admitted (age 50-59 years; %)**							
	1	126		25.3 (4.07; 3.54-38.4)		16.58	
	2	186		22.5 (2.57; 21.0-39.1)		6.61	
	3	201		22.1 (1.21; 9.76-27.2)		1.47	
	4	138		20.6 (0.64; 19.4-21.8)		0.41	
	5	126		19.8 (0.19; 19.6-20.1)		0.03	
**COVID-19 hospitalized admitted (age 60-69 years; %)**							
	1	125		17.46 (2.73; 10.5-24.3)		7.47	
	2	186		17.32 (1.91; 14.9-28.1)		3.64	
	3	202		17.11 (1.03; 7.65-22.5)		1.06	
	4	138		15.66 (0.29; 15.3-16.7)		0.08	
	5	126		15.13 (0.07; 15.1-15.3)		0.01	
**COVID-19 hospitalized admitted (age 70-79 years; %)**							
	1	126		10.17 (1.98; 4.56-16.3)		3.91	
	2	183		9.90 (1.16; 8.08-13.9)		1.33	
	3	202		10.6 (3.61; 5.44-60.5)		13.04	
	4	138		9.63 (0.08; 9.47-9.85)		0.01	
	5	126		9.55 (0.07; 9.45-9.63)		0.00	
**COVID-19 hospitalized admitted (age 80-89 years; %)**							
	1	125		5.498 (1.54; 0.88-9.43)		2.38	
	2	183		4.77 (0.45; 3.74-7.16)		0.20	
	3	202		5.01 (0.57; 2.41-7.16)		0.32	
	4	138		4.74 (0.19; 4.44-4.92)		0.04	
	5	126		4.87 (0.12; 4.69-5.01)		0.01	

^a^Values, n represents the number of observations or number of records pooled for the mean sample calculations. All other values represent the absolute descriptive statistical values.

#### COVID-19 Hospital Deaths Age Profile in South Africa

Table 8 shows the descriptive statistics for the COVID-19 hospital death age profiles for the first, second, third, fourth, and fifth COVID-19 epidemic wave periods in South Africa. Table 8 also shows the cumulative COVID-19 death age risk ratio, with age groups of 0 to 9 years as the reference.

**Figure 7:**
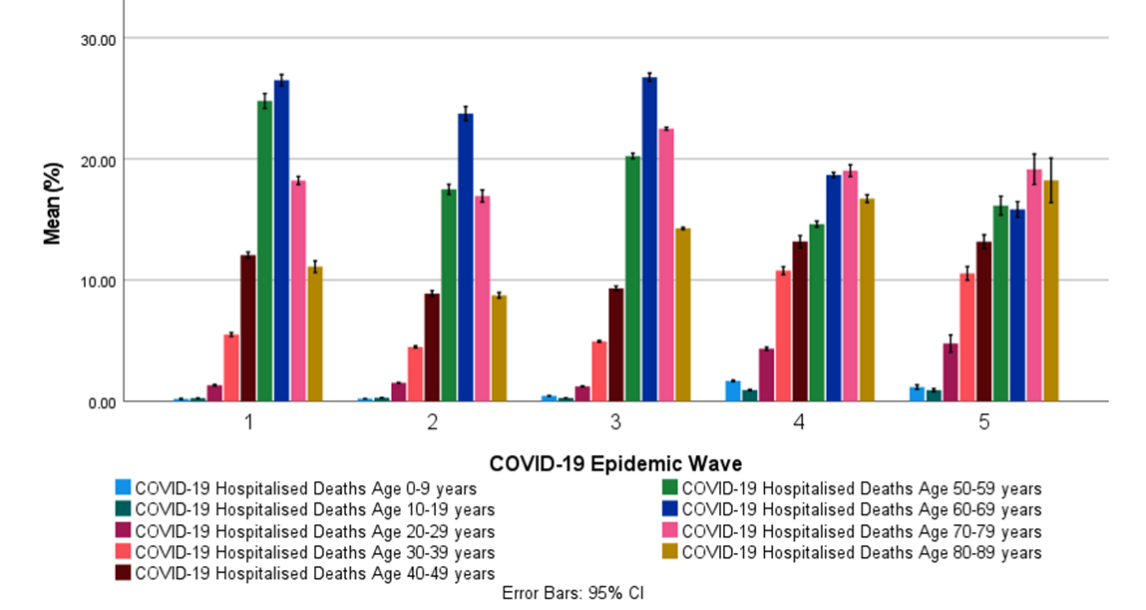
The mean COVID-19 hospitalized death age profile (with 95% CI error bars) in the first, second, third, fourth, and fifth COVID-19 epidemic wave period in South Africa.

Figure 7 shows the mean COVID-19 hospital death age profiles in the first, second, third, fourth, and fifth COVID-19 epidemic wave periods in South Africa. Figure 7 shows that most COVID-19 hospitalized deaths in South Africa in the first, second, third, fourth, and fifth COVID-19 epidemic wave periods were in the ages of 50 to 59 years (15.8%-24.8%), 60 to 69 years (15.9%-29.5%), and 70 to 79 years (16.6%-20.7%). COVID-19 hospitalized deaths in the age groups of 0 to 29 years were relatively low (0.227%-4.89%). Figure 7 also shows that the COVID-19 hospitalized death age profiles for the first, second, and third COVID-19 epidemic wave periods were similar in distribution, while the distributions in the fourth and fifth COVID-19 epidemic waves were also similar. The mean COVID-19 hospitalized deaths in the fourth and fifth COVID-19 epidemic waves for the age groups of 0 to 49 years and 80 to 89 years were significantly higher than those observed in the first, second, and third COVID-19 epidemic waves, while the age groups of 50 to 69 years were significantly lower than the respective COVID-19 epidemic waves. General trends show an increase in COVID-19 hospitalized deaths in the age groups of 0 to 49 years and 80 to 89 years after each consecutive COVID-19 epidemic wave and a decrease in the age groups of 50 to 79 years. The cumulative risk of death in COVID-19 hospitalized deaths increased with increasing age groups. The cumulative risk of death in COVID-19 hospitalized deaths in the age groups above 40 years was significantly lower in the fourth and fifth COVID-19 epidemic waves when compared with prior COVID-19 epidemic waves.

Paired *t* tests of the COVID-19 hospital admission age profile between the COVID-19 epidemic waves for the age groups of 10 to 39 years and 50 to 89 years showed statistically significant differences at a 95% CI between the respective COVID-19 epidemic periods with *P<*.001 (Table S11 in Multimedia Appendix 1). Paired *t* tests of the COVID-19 hospital admission age profile between the COVID-19 epidemic waves for the age groups of 40 to 49 years showed statistically significant differences at a 95% CI between the respective COVID-19 epidemic periods with *P<*.001, except for the paired *t* tests among the first, fourth, and fifth COVID-19 epidemic waves. The differences were statistically insignificant with *P*=.34 and *P=*.32 (Table S11 in Multimedia Appendix 1).

**Table 8:** Statistical sample number (n), range, mean, SD of COVID-19 hospitalized deaths in the ages of 0 to 9, 10 to 19, 20 to 29, 30 to 39, 40 to 49, 50 to 59, 60 to 69, 70 to 79, and 80 to 89 years in the first, second, third, fourth, and fifth COVID-19 epidemic wave periods in South Africa (descriptive statistics).

Parameter and COVID-19 epidemic wave		Values, n^a^	Values, mean (SD; range)	Variance	CFARR^b,c^
**COVID-19 hospitalized deaths (age 0-9 years; %)**					
	1	126	0.227 (0.154; 0-1.15)	0.024	Reference 1
	2	174	0.23 (0.082; 0-0.56)	0.007	Reference 2
	3	198	0.837 (5.3; 0.12-75)	28.1	Reference 3
	4	138	1.88 (1.043; 0.16-7.85)	1.087	Reference 4
	5	126	1.23 (1.193; 0.08-7.69)	1.424	Reference 5
**COVID-19 hospitalized deaths (age 10-19 years; %)**					
	1	126	0.298 (0.17; 0-1.70)	0.029	1.31
	2	182	0.309 (0.109; 0.23-1.45)	0.012	1.35
	3	201	0.273 (0.076; 0-0.36)	0.006	0.33
	4	134	1.01 (0.595; 0-5.25)	0.354	0.54
	5	126	0.94 (0.711; 0.03-5.56)	0.506	0.76
**COVID-19 hospitalized deaths (age 20-29 years; %)**					
	1	126	1.57 (0.65; 0-7.04)	0.422	6.90
	2	183	1.58 (0.442; 1.14-4.93)	0.195	6.86
	3	200	1.22 (0.26; 0.00-1.61)	0.068	1.45
	4	138	4.68 (2.31; 0.94-21.45)	5.34	2.49
	5	126	4.89 (4.21; 0.34-22.22)	17.73	3.99
**COVID-19 hospitalized deaths (age 30-39 years; %)**					
	1	126	6.02 (2.01; 1.03-26.82)	4.02	26.5
	2	183	4.51 (0.828; 2.93-8.75)	0.686	19.6
	3	202	5.08 (1.51; 3.07-25)	2.27	6.06
	4	138	11.9 (6.29; 4.05-51.78)	39.52	6.33
	5	126	10.6 (3.23; 0.92-25.93)	10.45	8.65
**COVID-19 hospitalized deaths (age 40-49 years; %)**					
	1	126	13.7 (4.8; 7.22-63.29)	23.04	60.2
	2	183	8.78 (1.87; 4.19-13.75)	3.50	38.2
	3	202	9.19 (1.59; 0-10.88)	2.54	11.0
	4	138	14.2 (6.54; 11.17-59.95)	42.82	7.57
	5	125	13.2 (3.26; 1.58-18.73)	10.64	10.7
**COVID-19 hospitalized deaths (age 50-59 years; %)**					
	1	76	24.8 (2.68; 14.78-37.50)	7.21	109
	2	179	17.1 (3.15; 9.29-19.43)	9.93	74.6
	3	202	20.3 (1.7; 17.14-25)	2.88	24.2
	4	138	15.8 (7.21; 12.03-70.04)	52.0	8.42
	5	126	16.1 (4.38; 1.90-25.78)	19.2	13.1
**COVID-19 hospitalized deaths (age 60-69 years; %)**					
	1	125	29.5 (4.11; 23.80-39.86)	16.9	130
	2	183	23.5 (4.62; 13.13-36.67)	21.32	103
	3	201	26.9 (2.6; 24.81-36.97)	6.78	32.1
	4	134	18.5 (2.05; 0-24.09)	4.22	9.82
	5	126	15.9 (3.61; 1.49-22.22)	13.01	12.9
**COVID-19 hospitalized deaths (age 70-79 years; %)**					
	1	125	20.7 (3.33; 11.75-25.45)	11.11	91.3
	2	183	16.6 (3.7; 8.28-22.28)	13.7	72.4
	3	202	22.6 (2.18; 15.13-50)	4.74	27.0
	4	138	20.1 (10.5; 6.67-102)	110	10.7
	5	126	19.1 (7.1; 1.56-91.57)	50	15.6
**COVID-19 hospitalized deaths (age 80-89 years; %)**					
	1	125	13.5 (3.44; 2.16-18.18)	11.8	59.6
	2	183	8.77 (2.22; 4.95-27.54)	4.93	38.2
	3	202	14.3 (1.03; 11.74-25)	1.06	17.1
	4	136	17.7 (8.8; 5.13-87.65)	77.5	9.39
	5	126	18.2 (10.38; 1.11-92.06)	107.8	14.8

^a^Values, n represents the number of observations or records pooled for the mean sample calculations. All other values represent absolute descriptive statistical values.

^b^CFARR: cumulative COVID-19 death age risk ratio.

^c^CFARR with COVID-19 epidemic wave references, reference 1, reference 2, reference 3, reference 4, and reference 5 as the mean COVID-19 hospitalized deaths (age 0 to 9 years; %) for the first, second, third, fourth, and fifth COVID-19 epidemic wave period in South Africa, respectively.

#### COVID-19 CFR, DR, and ECDR in South Africa

Table 9 shows the descriptive statistics for the COVID-19 hospital CFR, hospital DR, natural deaths, excess natural deaths, weekly reported COVID-19 deaths, and weekly unreported ECDR for the first, second, third, fourth, and fifth COVID-19 epidemic wave periods in South Africa. Paired *t* tests of the CFR between the COVID-19 epidemic waves showed statistically significant differences at a 95% CI between the respective COVID-19 epidemic periods with *P<*.001 (Table S12 in Multimedia Appendix 1). Except for the paired *t* tests among the third, fourth, and fifth COVID-19 epidemic waves. Paired *t* tests of the DR between the COVID-19 epidemic waves showed no statistically significant differences at a 95% CI between the respective COVID-19 epidemic periods with lowest *P*=.27 (Table S12 in Multimedia Appendix 1). Except for the paired *t* tests between the third and fourth COVID-19 epidemic periods. Paired *t* tests of the ECDR between the COVID-19 epidemic waves showed no statistically significant differences at a 95% CI between the respective COVID-19 epidemic periods with lowest *P*=.69 (Table S12 in Multimedia Appendix 1). Paired *t* tests of the weekly COVID-19 deaths and excess deaths between the COVID-19 epidemic waves showed statistically significant differences at a 95% CI between the respective COVID-19 epidemic periods with *P<*.001 (Table S12 in Multimedia Appendix 1).

**Table 9:** Statistical sample number (n), range, mean, SD of COVID-19 hospital case fatality rate (CFR), hospital discharge rate (DR), weekly natural deaths, excess natural deaths, reported COVID-19 deaths, and the weekly unreported excess deaths (natural) to COVID-19 death ratio (ECDR) in the first, second, third, fourth, and fifth COVID-19 epidemic wave periods in South Africa (descriptive statistics).

Parameter and COVID-19 epidemic wave		Values, n^a^	Values, mean (SD; range)
**CFR**			
	1	121	2.06 (1.10; 0 to 6.9)
	2	180	2.33 (1.59; 0 to 12.9)
	3	199	1.76 (1.18; 0.07 to 10.1)
	4	129	1.63 (7.57; 0 to 86.3)
	5	117	0.99 (1.72; 0 to 18.1)
**DR**			
	1	121	8.40 (4.89; 0 to 42.5)
	2	180	8.03 (8.62; 0 to 77.6)
	3	200	6.11 (2.90; 0.19 to 16.0)
	4	137	9.30 (7.70; 0 to 63.2)
	5	118	10.1 (10.7; 0 to 111.9)
**ECDR**			
	1	22	1.88 (2.34; −0.37 to 6.43)
	2	30	1.99 (1.38; 0.30 to 4.25)
	3	29	2.78 (1.92; 0.53 to 8.96)
	4	18	4.81 (5.58; −0.43 to 17.64)
	5	18	13.3 (8.48; 5.34 to 30.30)
**Weekly excess deaths**			
	1	22	2134 (2155; 35 to 6676)
	2	30	3822 (4277; 752 to 16,123)
	3	29	3856 (2837; 834 to 10,339)
	4	19	1655 (954; 832 to 3571)
	5	20	1197 (492; 383 to 2055)
**Weekly natural deaths**			
	1	36	9975 (2387; 7819 to 15,865)
	2	30	11,941 (4341; 9041 to 24,215)
	3	29	12,751 (3262; 8863 to 19,959)
	4	19	9558 (1168; 8495 to 11,891)
	5	20	10,070 (624; 8945 to 11,197)
**Weekly COVID-19 deaths**			
	Baseline	4	7884 (554; 7454 to 8662)
	1	35	469 (574; 0 to 2042)
	2	30	1258 (1123; 324 to 3942)
	3	29	1228 (902; 155 to 2916)
	4	18	577 (456; 85 to 1674)
	5	20	101 (71; 0 to 232)

^a^Values, n represents the number of observations or number of records pooled for the mean sample calculations. All other values represent the absolute descriptive statistical values.

## Discussion

### Principal Findings

The values of the Pearson correlation coefficients obtained between the daily COVID-19 tests and cases in this study indicate a strong positive association between daily COVID-19 tests and cases in the 5 COVID-19 epidemic waves in South Africa. The *F* test values and standardized coefficients obtained between the respective parameters using ANOVA and the Univariate General Linear Model indicated that the residual error between the linear predicted values and the actual values was relatively small, showing a high degree of linearity. On the basis of the Probability Theory, an increase in testing results in an increased probability of the outcome of detection of a positive COVID-19 test. In this study, COVID-19 transmissibility was measured based on the magnitude of the mean and variance of daily COVID-19 positive tests (COVID-19 detection rate). Considering the linear positive association between daily COVID-19 tests and cases, the COVID-19 detection rate normalized the variance between the respective parameters. The mean daily positive COVID-19 tests in South Africa’s first, second, third, fourth, and fifth COVID-19 epidemic wave periods were 11.5% (SD 8.58%), 11.5% (SD 8.45%), 13.3% (SD 9.72%), 13.1% (SD 9.91%), and 14.3% (SD 8.49%), respectively. The mean daily positive COVID-19 test results in South Africa’s first and second COVID-19 epidemic waves showed no significant difference at a 95% CI with *P=*.97. The mean daily positive COVID-19 tests in South Africa’s third, fourth, and fifth COVID-19 epidemic waves were 15.7%, 18.4%, and 24.3% higher than those of the first and second COVID-19 epidemic wave periods, respectively, with statistically significant differences at a 95% CI. These results suggest that the COVID-19 transmission rates in the first and second COVID-19 epidemic waves in South Africa were similar, while the COVID-19 transmission rate was higher in the third, fourth, and fifth COVID-19 epidemic waves than in the first and second waves.

The negative mean change in movement from baseline in retail and recreation, grocery and pharmacy, parks, transit stations, and workspaces gradually decreased during each consecutive COVID-19 epidemic wave as NPIs in South Africa were relaxed. The mean change in movement from baseline in residential locations was positive for the 5 COVID-19 epidemic waves. By the fourth and fifth COVID-19 epidemic waves, the mean change in movement from the baseline in all South African locations was positive, indicating greater movement than that observed before the COVID-19 pandemic (baseline, median values of movement in the respective locations from January 3 to February 6, 2020). The daily effective contact rate is the average number of adequate contacts per infective per day; it is directly proportional to the reproductive number [43]. Mabuka et al [38] showed through stochastic COVID-19 modeling that adjusting the NPIs by 1 Alert Level in South Africa translated into a reduction in the effective SARS-CoV-2 daily contact number by 4.13% to 14.6%. Thus, the relaxation of NPIs in South Africa after each consecutive COVID-19 epidemic wave could have possibly contributed to the increase in COVID-19 transmissibility in the COVID-19 epidemic waves.

The emerging dominant SARS-CoV-2 lineages in the South African SARS-CoV-2 genotypes collected during the first, second, third, fourth, and fifth COVID-19 epidemic waves were alpha (B.1.1.54, B.1.1.56, and C.1), beta (B.1.351), delta (B.1.617.2), and omicron B.1.1.529 and omicron BA.4, BA.2*, BA.5, BA.4, and BA.5.* SARS-CoV-2 variants or lineages, respectively. The beta, delta, and omicron SARS-CoV-2 lineages had major mutations in the spike protein. The beta (B.1.351) SARS-CoV-2 variant had 8 of its 17 mutations in the spike protein at Δ69-70 deletion, Δ144 deletion, N501Y, A570D, P681H, T716I, S982A, and D1118H [44-46]. The delta (B.1.617.2) SARS-CoV-2 variant had 10 mutations in the spike protein at T19R (G142D*), 156del, 157del, R158G, L452R, T478K, D614G, P681R, and D950N [47]. The omicron SARS-CoV-2 variant (B.1.1.529 lineage) had at least 34 mutations (30 amino acid substitutions, 3 small deletions, and 1 small insertion) in its genome. In total, 15 of the 30 amino acid substitutions in the omicron SARS-CoV-2 variant were in the receptor-binding domain. The key amino acid substitutions in the spike (S) protein were at A67V, del69-70, T95I, del142-144, Y145D, del211, L212I, ins214EPE, G339D, S371L, S373P, S375F, K417N, N440K, G446S, S477N, T478K, E484A, Q493R, G496S, Q498R, N501Y, Y505H, T547K, D614G, H655Y, N679K, P681H, N764K, D796Y, N856K, Q954H, N969K, L981F [48]. SARS-CoV-2 during infection binds to human angiotensin-converting enzyme (ACE) 2 receptors through the SARS-CoV-2 spike protein (S1). Some of the mutations observed in these lineages showed increased affinity by the spike protein in SARS-CoV-2 to the human ACE 2 receptors [49,50]. Thus, the increased COVID-19 transmission rate in the third, fourth, and fifth COVID-19 epidemic waves in South Africa could have been also a result of the mutations in the detected SARS-CoV-2 lineages in the respective COVID-19 epidemic waves.

The values of the Pearson, standardized coefficients, and coefficient of determination obtained between the number of COVID-19 active and hospital admission cases in this study indicated a strong linear positive association between COVID-19 active and hospital admission cases in the 5 COVID-19 epidemic waves in South Africa. This correlation was also well demonstrated by stochastic COVID-19 epidemiological models [38,51,52]. Considering this linear positive association between COVID-19 active and hospital admission cases, COVID-19 HA was used to understand the impact of SARS-CoV-2 lineages and COVID-19 vaccination on COVID-19 hospitalization in South Africa. The mean COVID-19 daily HA in South Africa’s first, second, third, fourth, and fifth COVID-19 epidemic wave periods were 6.80% (SD 1.82%), 18.7% (SD 7.08%), 16.5% (SD 5.56%), 9.84% (SD 4.48%), and 11.6% (SD 11.6%), respectively. The second COVID-19 epidemic wave period in South Africa had the highest number of COVID-19 HA, followed by the third COVID-19 epidemic wave period. The COVID-19 HA decreased during the first half period of the third, fourth, and fifth COVID-19 epidemic waves (Multimedia Appendices 4-6). The first half period of the COVID-19 epidemic wave is the period from the first case to the peak of the epidemic wave.

Most COVID-19 hospitalized cases in South Africa were hospitalized in the general ward (60.0%-79.1%). Patients with COVID-19 on oxygen were the second-largest admission status (11.2%-16.8%), followed by patients with COVID-19 in the ICU (8.07%-16.7%). The general trend showed a decrease in patients admitted on oxygen, in the ICU, and on a ventilator in the fourth and fifth COVID-19 epidemic waves compared with previous COVID-19 epidemic waves. The patients in high care remained relatively similar, with a mean range of 5.65% to 7.93% throughout the COVID-19 epidemic in South Africa. Most COVID-19 hospitalized cases in South Africa’s first, second, third, and fourth COVID-19 epidemic wave periods were in the ages of 40 to 49 years (16.8%-20.4%) and 50 to 59 years (19.8%-25.3%). Patients admitted owing to COVID-19 in the age groups of 0 to 19 years were relatively low (1.98%-4.59%) and highest in the fourth and fifth COVID-19 epidemic wave periods. In general, COVID-19 hospital admissions in South Africa for the age groups between 0 and 29 years increased after each consecutive COVID-19 epidemic wave, whereas for age groups between 30 and 79 years, they decreased. Most COVID-19 hospitalized deaths in South Africa in the first, second, third, fourth, and fifth COVID-19 epidemic wave periods were in the ages of 50 to 59 years (15.8%-24.8%), 60 to 69 years (15.9%-29.5%), and 70 to 79 years (16.6%-20.7%). COVID-19 hospitalized deaths in the age groups of 0 to 29 years were relatively low (0.227%-4.89%). The worldwide incidence of COVID-19 deaths in children has been reported to be low [8]. The cumulative risk of death in COVID-19 hospitalized deaths increased with increasing age groups. The cumulative risk of death in hospitalized COVID-19 deaths in the age groups above 40 years was significantly lower in the fourth and fifth COVID-19 epidemic waves when compared with prior COVID-19 epidemic waves. General trends show an increase in COVID-19 hospitalized deaths in the age groups between 0 and 49 years and 80 and 89 years after each consecutive COVID-19 epidemic wave and a decrease in the age groups between 50 and 79 years.

By the end of the fifth COVID-19 epidemic wave, 20,323,729 COVID-19 vaccine doses had been administered in South Africa. The COVID-19 vaccines administered in South Africa were Pfizer-BioNTech (Comirnaty) and Johnson & Johnson or Janssen COVID-19 vaccines. The percentage of the population per age group in South Africa who had received at least 1 dose of the COVID-19 vaccine by the end of the fifth COVID-19 epidemic wave was 32.8%, 52.9%, 66%, and 71.6% of the total population in the age groups of 18 to 34 years, 35 to 49 years, 50 to 59 years, and above 60 years, respectively. The COVID-19 vaccination rate in South Africa had dropped drastically in all age groups since the third COVID-19 epidemic wave. The values of the Spearman correlation coefficients obtained between the daily cumulative COVID-19 vaccinated people and COVID-19 HA in the half period of the third, fourth, and fifth COVID-19 epidemic waves in this study indicated a strong negative monotonic association between the cumulative COVID-19 vaccinated people and COVID-19 HA. The Spearman correlation coefficients between daily cumulative COVID-19 vaccinated people and the percentage of hospitalized age groups of 18 to 34 years showed a significantly strong positive monotonic association, while the age groups of 50 years to above 60 years showed a significantly strong negative monotonic association. The values of the Spearman correlation coefficients obtained between the daily cumulative COVID-19 vaccinated people and change in daily COVID-19 cases in the half period of the third, fourth, and fifth COVID-19 epidemic waves in this study indicate a weak monotonic association between the daily cumulative COVID-19 vaccinated people and the change in daily COVID-19 cases. These results suggest that COVID-19 vaccination had an association with the reduction in COVID-19 hospital admission. Pfizer-BioNTech (Comirnaty) and Johnson & Johnson or Janssen COVID-19 vaccines have shown high efficacy against severe COVID-19 at 85% and 76.7%, respectively [53,54].

The mean COVID-19 hospital CFR in South Africa’s first, second, third, fourth, and fifth COVID-19 epidemic wave periods were 2.06% (SD 1.10%), 2.33% (SD 1.59%), 1.76% (SD 1.18%), 1.63% (SD 7.57%), and 0.99% (SD 1.72%), respectively. The mean COVID-19 hospital DR in South Africa’s first, second, third, fourth, and fifth COVID-19 epidemic wave periods were 8.40% (SD 4.89%), 8.03% (SD 8.62%), 6.11% (SD 2.90%), 9.30% (SD 7.70%), and 10.05% (SD 10.73%), respectively. The CFRs in the third, fourth, and fifth COVID-19 epidemic waves in South Africa were lower than those observed in the first and second COVID-19 epidemic waves. The mean ECDR in South Africa’s first, second, third, fourth, and fifth COVID-19 epidemic wave periods was 1.88% (SD 2.34%), 1.99% (SD 1.38%), 2.78% (SD 1.92%), 4.81% (SD 5.58%) and 13.3% (SD 8.48%), respectively. The ECDR values obtained in this study suggest that there was a relatively high number of deaths related to the COVID-19 pandemic which occurred outside South African hospitals and were unreported. Paired *t* tests of the ECDR between the COVID-19 epidemic waves showed statistically significant indifferences at 95% CI, indicating that the COVID-19 death rate occurring outside South African hospitals was similar in the 5 COVID-19 epidemic waves. According to the NICD, most hospitalized cases in the third, fourth, and fifth COVID-19 epidemic waves were mostly patients unvaccinated against COVID-19 (66.4%) [55,56].

### Conclusions

The relaxation of COVID-19 NPI health policies in South Africa and the evolution of SARS-CoV-2 were associated with increased COVID-19 transmission and severity in the South African population. COVID-19 vaccination in South Africa was strongly associated with a decrease in COVID-19 hospitalizations and severity in South Africa.

## Appendix

Multimedia Appendix 1 SARS-CoV-2 lineages identified in genome samples and COVID-19 nonpharmacuetical intervention policies implemented in the South African COVID-19 epidemic waves; Pearson and Spearman correlation coefficients, ANOVA, univariate general linear model coefficients, and paired samples *t* tests for South African COVID-19 epidemiological data.

Multimedia Appendix 2 Mean change in movement from baseline in retail and recreation, grocery and pharmacy, parks, transit stations, workplaces, and residences during the implementation of South African COVID-19 nonpharmacuetical intervention policies.

Multimedia Appendix 3 Maximum number of people with at least 1 dose of COVID-19 vaccine administered per total population age group during the first, second, third, fourth, and fifth COVID-19 epidemic wave in South Africa.

Multimedia Appendix 4 COVID-19 hospital-to-active cases and vaccinated people in the first half period of the third COVID-19 epidemic wave.

Multimedia Appendix 5 COVID-19 hospital-to-active cases and vaccinated people in the first half period of the fourth COVID-19 epidemic wave.

Multimedia Appendix 6 COVID-19 hospital-to-active cases and vaccinated people in the first half period of the fifth COVID-19 epidemic wave.

## Abbreviations

ACE: angiotensin-converting enzyme
CFR: case fatality rate
DR: discharge rate
ECDR: excess deaths (natural) to COVID-19 death ratio
HA: hospital-to-active cases
ICU: intensive care unit
NICD: National Institute for Communicable Diseases
NPI: nonpharmaceutical intervention
VOC: variant of concern
VOI: variant of interest
WHO: World Health Organization
