# Supplementary material for: The Impact of SARS-CoV-2 Lineages (Variants) and COVID-19 Vaccination on the COVID-19 Epidemic in South Africa: Regression Study
Source: JMIRx Med. 2023 Jul 3;4:e34598. doi: 10.2196/34598 (PMC10337479; doi:10.2196/34598)
Supplement: Multimedia Appendix 1 [file xmed_v4i1e34598_app1.docx]

# Appendix

Table S 1 SARS-CoV-2 lineages identified in genome samples obtained from patients with laboratory-confirmed COVID-19 for the four observed COVID-19 epidemic wave periods in South Africa. Data extracted from [18,20,21,25–27,41]

| **SARS-CoV-2 Lineage Cluster** | 1 | 2 | 3 | 4 | 5 |
| --- | --- | --- | --- | --- | --- |
| **Epidemic Period** | 2020/03/05-2020/09/30 | 2020/10/01-2021/04/26 | 2021/04/27-2021/11/14 | 2021/11/15-2022/04/01 | 2022/04/02-2022/07/31 |
| **Genome Samples** | 1365 | 2 573 | 10230 | 5778 | 5778 |
| **Genome Sampling Period** | 2020/03/06-2020/08/26 | 2020/03/05-2020/12/10 | 2021/05-2021/11 | 2021/11-2022/04 | 2022/04-2022/07 |
| **SARS-CoV-2 Lineages in Samples** | A, B, B.1, B.1.1, B.1.1.1, B.1.1.10, B.1.1.26, B.1.1.34, B.1.1.35, B.1.1.40, B.1.1.52, B.1.1.53, B.1.1.54, B.1.1.56, B.1.1.57, B.1.1.62, B.1.1.64, B.1.1.66, B.1.1.67, B.1.1.75, B.1.106, B.1.132, B.1.133, B.1.140, B.1.144, B.1.23, B.1.35, B.1.5, B.1.5.16, B.1.5.19, B.1.5.22, B.1.79, B.1.8, B.1.98, B.2, B.2.1, B.2.2, B.3, B.6, B.9, C.1, C.2 | A, B, B.1, B.1.1, B.1.1.1, B.1.1.10, B.1.109, B.1.1.117, B.1.1.119, B.1.1.156, B.1.1.159, B.1.1.163, B.1.1.2, B.1.1220, B.1.1.252, B.1.1.254, B.1.1.316,B.1.1.34, B.1.1.4, B.1.1.40, B.1.1.52, B.1.1.53, B.1.1.54, B.1.1.56, B.1.1.57, B.1.1.62, B.1.1.64, B.1.1.67, B.1.1.75, B.1.1.84, B.1.1.91, B.1.1.99, B.1.106, B.1.124, B.1.131, B.1.140, B.1.141, B.1.144, B.1.153, B.1.178, B.1.183, B.1.222, B.1.225, B.1.237, B.1.242, B.1.275, B.1.315, B.1.36, B.1.5, B.1.5.16, B.1.5.19, B.1.5.31, B.1.8, B.1.98, B.3, B.39, B.40, B.52, B.6, C.1, C.2, C.6, C.9, H.1 | B.1.351, B.1.617.1, B.1.617.2, B.1.525, B.1.1.7, C.1.2, Other (1.74 %), unassigned (5.20 %) | B.1.1.529, B.1.617.2, B.1.351, C.1.2 | BA.4, BA.2,BA.5, 21J, BA.1*, BA.4, BA.5.*, Other, Unassigned, C.1.2, BA.1, BA.3, BA.2, BA.2.75 |
| **SARS-CoV-2 Lineages in Epidemic Period** | A, B, B.1, B.1.1, B.1.1.1, B.1.1.10, B.1.1.26, B.1.1.34, B.1.1.35, B.1.1.40, B.1.1.52, B.1.1.53, B.1.1.54, B.1.1.56, B.1.1.57, B.1.1.62, B.1.1.64, B.1.1.66, B.1.1.67, B.1.1.75, B.1.106, B.1.132, B.1.133, B.1.140, B.1.144, B.1.23, B.1.35, B.1.5, B.1.5.16, B.1.5.19, B.1.5.22, B.1.79, B.1.8, B.1.98, B.2, B.2.1, B.2.2, B.3, B.6, B.9, C.1, C.2 | B.1,B.1.1,B.1.1.119, B.1.1.54, B.1.1.56, C.1, B.1.351, B.1.1.156, B.1.1.252, B.1.1.254, B.1.1.34, B.1.1.57, B.1.1.220, B.1.144, B.1.237, B.1.351, B.1.5, B.1.5.19, B.1.5.31, C.2, C.9, B.1.106 | B.1.351, B.1.617.1, B.1.617.2, B.1.525, B.1.1.7, C.1.2 | B.1.1.529, B.1.617.2, B.1.351, C.1.2 | BA.4, BA.2,BA.5, 21J, BA.1*, BA.4, BA.5.*, Other, Unassigned, C.1.2, BA.1, BA.3, BA.2, BA.2.75 |
| **SA Specific SARS-CoV-2 Lineages** | B.1.1.34, B.1.1.40, B.1.1.52, B.1.1.53, B.1.1.54, B.1.1.56, B.1.1.57, B.1.1.62, B.1.1.66, B.1.106, B.1.133, B.1.144, B.1.5.16, B.1.5.19, C.1, C.2 | B.1.1.34, B.1.1.54, B.1.1.56, C.1, B.1.351, B.1.1.57, B.1.106, B.1.144, B.1.5.19, C.2 | B.1.351, C.1.2 | B.1.351, C.1.2 | C.1.2 |
| **Emerging Dominant SARS-COV-2 Lineages Spatiotemporal Phylogeographic** | **B.1.106** (April 2020)**, B.1.1.54** (poor temporal signaling), **B.1.1.56** (mid-March 2020 (95% highest posterior density (HPD) 2020-02-15 to 2020-03-30)), **C.1** (early May 2020 (95% HPD 2020-04-24 to 2020-05-24)) | **B.1.351** (early August (95% highest posterior density ranging from the middle of July to the end of August 2020)) |  |  |  |
| **Emerging Dominant SARS-COV-2 Lineages in Samples (%)** | B.1.1.54, B.1.1.56, C.1 (42.1 % of total genomes) | B.1.351 (Approximately 90 % of Samples by End of November 2020) | B.1.617.2 (Approximately Over 65 % in June, 85 % in July and over 90 % of samples in August and September 2021) | B.1.1.529 (Approximately Over 82 % in November 2021, 96 % in December 2021 and 98.5 % of samples in January and February 2022), C.1.2 (absent in samples from January and February 2022.) | BA.4 (Aproximately 54% in April 2022),BA.2*(Aproximately 25% in April 2022,5% in May ,BA.5 (Aproximately 19% in April 2022), BA.4(Aproximately 64% in May 2022, 53% in June 2022, 28% in July 2022) ,BA.5.*(Aproximately 29% in May 2022, 39% in June 2022, 68% in July 2022) |

Table S 2: Pearson Correlation Coefficients, Coefficient of determination and P-values (Sig. (2-tailed)) between Daily COVID-19 Tests (Independent Variable) and Daily COVID-19 Cases (Dependent Variable) in the First, Second, Third, Fourth and Fifth COVID-19 Epidemic Wave in South Africa (Correlations)

| **Correlation^c^** | | | | | | |
| --- | --- | --- | --- | --- | --- | --- |
| Parameter | COVID-19 Epidemic Wave | Pearson Correlation | Coefficient of determination | Sig. (2-tailed) | 95% CI | |
|  |  |  |  |  | Lower | Upper |
| Daily COVID-19 Tests | 1 | 0.910 | 0.828 | *P<.*001 | 0.883 | 0.931 |
|  | 2 | 0.877 | 0.769 | *P<.*001 | 0.841 | 0.906 |
|  | 3 | 0.893 | 0.797 | *P<.*001 | 0.860 | 0.919 |
|  | 4 | 0.859 | 0.737 | *P<.*001 | 0.806 | 0.898 |
|  | 5 | 0.749 | 0.562 | *P<.*001 | 0.613 | 0.842 |
| ^a.^ Estimation is based on Fisher's r-to-z transformation. | | | | | | |
| ^b.^ Estimation of standard error is based on the formula proposed by Fieller, Hartley, and Pearson. | | | | | | |
| ^c.^ Dependent Variable: Daily COVID-19 Cases | | | | | | |

Table S 3: Sum of Squares, Degrees of Freedom (df), Mean Square, F values between Mean Square Regression and Residual (F), between Daily COVID-19 Tests (Independent Variable) and Daily COVID-19 Cases (Dependent Variable) in the First, Second, Third, Fourth and Fifth COVID-19 Epidemic Wave in South Africa **(Analysis of Variance (ANOVA)**)

| **ANOVA^a^** | | | | | | |
| --- | --- | --- | --- | --- | --- | --- |
| COVID-19 Epidemic Wave | | Sum of Squares | df | Mean Square | F | Sig. |
| 1 | Regression | 2,548,543,000 | 1 | 2,548,543,000 | 967 | *P<.*001^b^ |
|  | Residual | 529,940,020 | 201 | 2,636,518 |  |  |
|  | Total | 3,078,483,020 | 202 |  |  |  |
| 2 | Regression | 4,024,599,374 | 1 | 4,024,599,374 | 664 | *P<.*001^b^ |
|  | Residual | 1,206,837,402 | 199 | 6,064,510 |  |  |
|  | Total | 5,231,436,776 | 200 |  |  |  |
| 3 | Regression | 5,868,645,201 | 1 | 5,868,645,201 | 744 | *P<.*001^b^ |
|  | Residual | 1,490,659,713 | 189 | 7,887,088 |  |  |
|  | Total | 7,359,304,915 | 190 |  |  |  |
| 4 | Regression | 4,498,350,410 | 1 | 4,498,350,410 | 368 | *P<.*001^b^ |
|  | Residual | 1,601,724,113 | 131 | 12,226,902 |  |  |
|  | Total | 6,100,074,523 | 132 |  |  |  |
| 5 | Regression | 290,500,063 | 1 | 290,500,063 | 75.596069 | *P<.*001^b^ |
|  | Residual | 226,724,800 | 59 | 3,842,793 |  |  |
|  | Total | 517,224,863 | 60 |  |  |  |
| ^a.^ Dependent Variable: Daily COVID-19 Cases | | | | | | |
| ^b.^ Predictors: (Constant), Daily COVID-19 Tests | | | | | | |

Table S 4: Unstandardized Coefficients (B, Std. Error), Standardized Coefficients (Beta), t-calculated (t), P-value calculated (Sig.) and 95.0 % Confidence Interval for B between Daily COVID-19 Tests (Independent Variable) and Daily COVID-19 Cases (Dependent Variable) in the First, Second, Third, Fourth and Fifth COVID-19 Epidemic Wave in South Africa **(Coefficients**)

| **Coefficients^a^** | | | | | | |
| --- | --- | --- | --- | --- | --- | --- |
| COVID-19 Epidemic Wave | | Unstandardized Coefficients | | Standardized Coefficients | t | Sig. |
|  |  | B | Std. Error | Beta |  |  |
| 1 | (Constant) | -1878 | 202 |  | -9.28 | *P<.*001 |
|  | Daily COVID-19 Tests | 0.253 | 0.008 | 0.910 | 31.1 | *P<.*001 |
| 2 | (Constant) | -5490 | 421 |  | -13.1 | *P<.*001 |
|  | Daily COVID-19 Tests | 0.318 | 0.012 | 0.877 | 25.8 | *P<.*001 |
| 3 | (Constant) | -7647 | 561 |  | -13.6 | *P<.*001 |
|  | Daily COVID-19 Tests | 0.345 | 0.013 | 0.893 | 27.3 | *P<.*001 |
| 4 | (Constant) | -6338 | 697 |  | -9.09 | *P<.*001 |
|  | Daily COVID-19 Tests | 0.342 | 0.018 | 0.859 | 19.2 | *P<.*001 |
| 5 | (Constant) | -3413 | 850 |  | -4.0 | *P<.*001 |
|  | Daily COVID-19 Tests | 0.301 | 0.035 | 0.749 | 8.7 | *P<.*001 |
| ^a.^ Dependent Variable: Daily COVID-19 Cases | | | | | | |

Table S 5: Pearson Correlation Coefficients, Co-efficient of determination and P-values (Sig. (2-tailed)) between Number of Facilities Reporting to the NICD DATCoV System (Independent Variable), COVID-19 Active Cases (Covariant Variable) and COVID-19 Hospital Admitted Cases (Dependent Variable) in the First, Second, Third, Fourth and Fifth COVID-19 Epidemic Wave in South Africa **(Correlations**)

| **Correlation^c^** | | | | | | |
| --- | --- | --- | --- | --- | --- | --- |
| Parameter | COVID-19 Epidemic Wave | Pearson Correlation | Coefficient of determination | Sig. (2-tailed) | 95% CI | |
|  |  |  |  |  | Lower | Upper |
| COVID-19 Active Cases | 1 | 0.932 | 0.869 | *P<.*001 | 0.905 | 0.952 |
|  | 2 | 0.819 | 0.671 | *P<.*001 | 0.766 | 0.861 |
|  | 3 | 0.967 | 0.936 | *P<.*001 | 0.957 | 0.975 |
|  | 4 | 0.919 | 0.845 | *P<.*001 | 0.888 | 0.942 |
|  | 5 | 0.863 | 0.745 | *P<.*001 | 0.807 | 0.904 |
| Facilities Reporting to NICD DATCoV | 1 | 0.336 | 0.113 | *P<.*001 | 0.172 | 0.483 |
|  | 2 | 0.212 | 0.045 | *P=.*00355 | 0.071 | 0.345 |
|  | 3 | 0.385 | 0.148 | *P<.*001 | 0.261 | 0.497 |
|  | 4 | -0.249 | 0.062 | *P=.*00517 | -0.407 | -0.076 |
| ^a.^ Estimation is based on Fisher's r-to-z transformation. | | | | | | |
| ^b.^ Estimation of standard error is based on the formula proposed by Fieller, Hartley, and Pearson. | | | | | | |
| ^c.^ Dependent Variable: COVID-19 Hospital Admitted Cases | | | | | | |

Table S 6: Unstandardized Coefficients (B, Std. Error), Standardized Coefficients (Beta), t-calculated (t), P-value calculated (Sig.) and 95.0 % Confidence Interval for B between Number of Facilities Reporting to the NICD DATCoV System (Independent Variable), COVID-19 Active Cases (Covariant Variable) and COVID-19 Hospital Admitted Cases (Dependent Variable) in the First, Second, Third, Fourth and Fifth COVID-19 Epidemic Wave in South Africa **(Coefficients**)

| **Coefficients^a^** | | | | | | |
| --- | --- | --- | --- | --- | --- | --- |
| Epidemic_Wave | | Unstandardized Coefficients | | Standardized Coefficients | t | Sig. |
|  |  | B | Std. Error | Beta |  |  |
| 1 | (Constant) | 1807 | 122 |  | 14.8 | *P<.*001 |
|  | COVID-19 Active Cases | 0.036 | 0.001 | 0.932 | 28.7 | *P<.*001 |
| 2 | (Constant) | 3953 | 427 |  | 9.26 | *P<.*001 |
|  | COVID-19 Active Cases | 0.095 | 0.005 | 0.819 | 19.5 | *P<.*001 |
| 3 | (Constant) | 3252 | 195 |  | 16.7 | *P<.*001 |
|  | COVID-19 Active Cases | 0.095 | 0.002 | 0.967 | 53.9 | *P<.*001 |
| 4 | (Constant) | 2341 | 133 |  | 17.5 | *P<.*001 |
|  | COVID-19 Active Cases | 0.036 | 0.001 | 0.919 | 27.2 | *P<.*001 |
| 5 | (Constant) | 1416 | 58 |  | 24.6 | *P<.*001 |
|  | COVID-19 Active Cases | 0.029 | 0.002 | 0.863 | 18.0 | *P<.*001 |
| 1 | (Constant) | 2416 | 626 |  | 3.86 | *P<.*001 |
|  | Facilities Reporting to NICD DATCoV | 6.893 | 1.73 | 0.336 | 3.98 | *P<.*001 |
| 2 | (Constant) | -34186 | 15131 |  | -2.26 | *P=.*00355 |
|  | Facilities Reporting to NICD DATCoV | 71.295 | 24 | 0.212 | 2.95 | *P=.*00350 |
| 3 | (Constant) | -182252 | 32871 |  | -5.54 | *P<.*001 |
|  | Facilities Reporting to NICD DATCoV | 293.998 | 50 | 0.385 | 5.91 | *P<.*001 |
| 4 | (Constant) | 1946322 | 353288 |  | 5.51 | *P<.*001 |
|  | Facilities Reporting to NICD DATCoV | -2918 | 531 | -0.426 | -5.49 | *P<.*001 |
| ^a.^ Dependent Variable: COVID-19 Hospital Admitted Cases | | | | | | |

Table S 7: Summary of COVID-19 NPI Policies implemented in South Africa during the First, Second, Third, Fourth and Fifth COVID-19 Epidemic Waves [42]

| **COVID-19 Epidemic Wave** | **COVID-19 NPI Policy Implemented** | **Period of Implementation** |
| --- | --- | --- |
| 1 | National Lockdown Alert Levels 5 | 26 March to 30 April 2020 |
|  | National Lockdown Alert Levels 4 | 1 May to 31 May 2020 |
|  | National Lockdown Alert Levels 3 | 1 June to 17 August 2020 |
|  | National Lockdown Alert Levels 2 | 18 August to 20 September 2020 |
| 2 | National Lockdown Adjusted Alert Levels 1 | 21 September to 28 December 2020 |
|  | National Lockdown Adjusted Alert Levels 3 | 29 December 2020 to 28 February 2021 |
|  | National Lockdown Adjusted Alert Levels 1 | 1 March to 30 May 2021 |
| 3 | National Lockdown Adjusted Alert Levels 2 | 31 May to 15 June 2021 |
|  | National Lockdown Adjusted Alert Levels 3 | 16 June to 27 June 2021 |
|  | National Lockdown Adjusted Alert Levels 4 | 28 June to 25 July 2021 |
|  | National Lockdown Adjusted Alert Levels 3 | 26 July to 12 September 2021 |
|  | National Lockdown Adjusted Alert Levels 2 | 13 September to 15 November 2021 |
| 4 | National Lockdown Adjusted Alert Levels 2 | 16 to 30 November 2021 |
|  | National Lockdown Adjusted Alert Levels 1 | 1 October 2021-1 April 2022 |
| 5 | National Lockdown Adjusted Alert Levels 1 | 1 April 2022-23 June 2022 |
|  | No National Lock Down | 23 June 2022- |

Table S 8: Spearman’s Correlation Coefficients and P-values (Sig. (2-tailed)) between Daily Cumulative COVID-19 Vaccinated People (Independent Variable) and Daily COVID-19 Cases, Change in Daily COVID-19 Cases, Change in Daily COVID-19 Deaths, HA, CFR, DR, COVID-19 Hopistalised Admitted and Deaths profiles (%) (Dependent Variable) in the half period of the Third, Fourth and Fifth COVID-19 Epidemic Wave in South Africa **(Correlations**)

| **Correlations Spearman's rho^c^** | | | | | |
| --- | --- | --- | --- | --- | --- |
| Parameter | COVID-19 Epidemic Wave | Spearman's rho | Significance(2-tailed) | 95 % CI Lower | 95 % CI Upper |
| Daily COVID-19 Cases | 3 | 0.930 | *P<.*001 | 0.890 | 0.956 |
|  | 4 | 0.842 | *P<.*001 | 0.713 | 0.916 |
|  | 5 | 0.811 | *P<.*001 | 0.673 | 0.895 |
| Change in Daily COVID-19 Cases | 3 | 0.031 | *P=.*79 | -0.207 | 0.266 |
|  | 4 | -0.014 | *P=.*93 | -0.341 | 0.316 |
|  | 5 | -0.077 | *P=.*62 | -0.374 | 0.233 |
| Change in Daily COVID-19 Deaths | 3 | -0.037 | *P=.*76 | -0.271 | 0.201 |
|  | 4 | 0.019 | *P=.*91 | -0.303 | 0.337 |
|  | 5 | 0.003 | *P=.*98 | -0.302 | 0.308 |
| HA | 3 | -0.983 | *P<.*001 | -0.989 | -0.972 |
|  | 4 | -0.852 | *P<.*001 | -0.921 | -0.731 |
|  | 5 | -0.917 | *P<.*001 | -0.955 | -0.850 |
| CFR | 3 | 0.380 | *P<.*001 | 0.160 | 0.565 |
|  | 4 | 0.192 | *P=.*25 | -0.150 | 0.494 |
|  | 5 | 0.264 | *P=.*0877 | -0.049 | 0.529 |
| DR | 3 | 0.318 | *P=.*00607 | 0.088 | 0.516 |
|  | 4 | 0.425 | *P=.*00622 | 0.122 | 0.656 |
|  | 5 | 0.251 | *P=.*0997 | -0.058 | 0.517 |
| COVID-19 Hospitalised Admitted Age 18-34 years (%) | 3 | -0.922 | *P<.*001 | -0.951 | -0.876 |
|  | 4 | 0.978 | *P<.*001 | 0.958 | 0.989 |
|  | 5 | 0.773 | *P<.*001 | 0.612 | 0.872 |
| COVID-19 Hospitalised Admitted Age 35-49 years (%) | 3 | -0.974 | *P<.*001 | -0.984 | -0.959 |
|  | 4 | 0.246 | *P=.*13 | -0.080 | 0.525 |
|  | 5 | -0.793 | *P<.*001 | -0.884 | -0.643 |
| COVID-19 Hospitalised Admitted Age 50-59 years (%) | 3 | -0.872 | *P<.*001 | -0.919 | -0.801 |
|  | 4 | -0.933 | *P<.*001 | -0.965 | -0.874 |
|  | 5 | -0.974 | *P<.*001 | -0.986 | -0.951 |
| COVID-19 Hospitalised Admitted Age Over 60 years (%) | 3 | 0.982 | *P<.*001 | 0.971 | 0.989 |
|  | 4 | -0.969 | *P<.*001 | -0.984 | -0.940 |
|  | 5 | -0.260 | *P=.*0877 | -0.524 | 0.049 |
| COVID-19 Hospitalised Deaths Age 18-34 years (%) | 3 | 0.186 | *P=.*12 | -0.056 | 0.408 |
|  | 4 | -0.300 | *P=.*0602 | -0.566 | 0.022 |
|  | 5 | -0.632 | *P<.*001 | -0.786 | -0.406 |
| COVID-19 Hospitalised Deaths Age 35-49 years (%) | 3 | -0.206 | *P=.*0805 | -0.422 | 0.032 |
|  | 4 | -0.890 | *P<.*001 | -0.942 | -0.796 |
|  | 5 | -0.047 | *P=.*76 | -0.347 | 0.262 |
| COVID-19 Hospitalised Deaths Age 50-59 years (%) | 3 | 0.117 | *P=.*32 | -0.121 | 0.343 |
|  | 4 | 0.204 | *P=.*21 | -0.124 | 0.492 |
|  | 5 | 0.102 | *P=.*51 | -0.209 | 0.395 |
| COVID-19 Hospitalised Deaths Age Over 60 years (%) | 3 | -0.251 | *P=.*0319 | -0.461 | -0.016 |
|  | 4 | 0.838 | *P<.*001 | 0.708 | 0.913 |
|  | 5 | 0.823 | *P<.*001 | 0.692 | 0.902 |
| ^a.^ Estimation is based on Fisher's r-to-z transformation. | | | | | |
| ^b.^ Estimation of standard error is based on the formula proposed by Fieller, Hartley, and Pearson. | | | | | |
| ^c.^ Independent Variable: Daily Cumulative COVID-19 Vaccinated People | | | | | |

Table S 9: Mean Paired Differences, Standard Deviation (Std.) Paired Differences, Standard Error of Mean (Std. Error Mean), 95% confidence interval (CI) if the Upper and Lower Difference, t-value, degrees of freedom (df) and P-value (Sig. (2-tailed) for the COVID-19 Hospitalised Cases in the General Ward, High Care, Intensive Care Unit, On Oxygen, On Ventilator in Pair 1 to Pair 7. **(Paired Samples T-Test**)

| **Paired Samples Test** | | | | | | |
| --- | --- | --- | --- | --- | --- | --- |
| **Sample T-test Pairing** | **Paired Variables** | **Paired Differences Mean** | **Paired Differences Std. Deviation** | **t** | **df** | **Sig. (2-tailed)** |
| Pair 1 | COVID-19 General Ward (%) 1 - COVID-19 General Ward (%) 2 | 16.2 | 2.43 | 74.5 | 124 | *P<.*001 |
| Pair 2 | COVID-19 General Ward (%) 1 - COVID-19 General Ward (%) 3 | 10.1 | 3.42 | 32.8 | 122 | *P<.*001 |
| Pair 3 | COVID-19 General Ward (%) 1 - COVID-19 General Ward (%) 4 | -6.35 | 2.16 | -21.0 | 50 | *P<.*001 |
| Pair 4 | COVID-19 General Ward (%) 1 - COVID-19 General Ward (%) 5 | -3.35 | 2.40 | -9.66 | 47 | *P<.*001 |
| Pair 5 | COVID-19 General Ward (%) 2 - COVID-19 General Ward (%) 3 | 1.51 | 12.6 | 1.64 | 187 | *P=.*103 |
| Pair 6 | COVID-19 General Ward (%) 3 - COVID-19 General Ward (%) 4 | -21.1 | 6.45 | -38.4 | 137 | *P<.*001 |
| Pair 7 | COVID-19 General Ward (%) 4 - COVID-19 General Ward (%) 5 | -0.01 | 8.08 | -0.02 | 126 | *P=.*99 |
| Pair 1 | COVID-19 High Care (%) 1 - COVID-19 High Care (%) 2 | 2.28 | 1.50 | 17.0 | 124 | *P<.*001 |
| Pair 2 | COVID-19 High Care (%) 1 - COVID-19 High Care (%) 3 | 1.97 | 1.43 | 15.3 | 122 | *P<.*001 |
| Pair 3 | COVID-19 High Care (%) 1 - COVID-19 High Care (%) 4 | 2.46 | 1.88 | 9.35 | 50 | *P<.*001 |
| Pair 4 | COVID-19 High Care (%) 1 - COVID-19 High Care (%) 5 | -0.61 | 1.18 | -3.60 | 47 | *P<.*001 |
| Pair 5 | COVID-19 High Care (%) 2 - COVID-19 High Care (%) 3 | -0.40 | 0.52 | -10.5 | 187 | *P<.*001 |
| Pair 6 | COVID-19 High Care (%) 3 - COVID-19 High Care (%) 4 | -1.10 | 1.14 | -11.4 | 137 | *P<.*001 |
| Pair 7 | COVID-19 High Care (%) 4 - COVID-19 High Care (%) 5 | -0.71 | 3.83 | -2.10 | 126 | *P=.*0379 |
| Pair 1 | COVID-19 ICU (%) 1 - COVID-19 ICU (%) 2 | 5.34 | 1.84 | 32.5 | 124 | *P<.*001 |
| Pair 2 | COVID-19 ICU (%) 1 - COVID-19 ICU (%) 3 | 4.91 | 1.85 | 29.3 | 122 | *P<.*001 |
| Pair 3 | COVID-19 ICU (%) 1 - COVID-19 ICU (%) 4 | 5.98 | 0.72 | 59.2 | 50 | *P<.*001 |
| Pair 4 | COVID-19 ICU (%) 1 - COVID-19 ICU (%) 5 | 4.20 | 1.91 | 15.2 | 47 | *P<.*001 |
| Pair 5 | COVID-19 ICU (%) 2 - COVID-19 ICU (%) 3 | -1.51 | 2.01 | -10.3 | 187 | *P<.*001 |
| Pair 6 | COVID-19 ICU (%) 3 - COVID-19 ICU (%) 4 | 4.50 | 1.82 | 29.0 | 137 | *P<.*001 |
| Pair 7 | COVID-19 ICU (%) 4 - COVID-19 ICU (%) 5 | -1.79 | 1.57 | -12.8 | 126 | *P<.*001 |
| Pair 1 | COVID-19 On Oxygen (%) 1 - COVID-19 On Oxygen (%) 2 | -4.09 | 6.84 | -6.69 | 124 | *P<.*001 |
| Pair 2 | COVID-19 On Oxygen (%) 1 - COVID-19 On Oxygen (%) 3 | 2.81 | 6.79 | 4.60 | 122 | *P<.*001 |
| Pair 3 | COVID-19 On Oxygen (%) 1 - COVID-19 On Oxygen (%) 4 | 7.73 | 4.03 | 13.71 | 50 | *P<.*001 |
| Pair 4 | COVID-19 On Oxygen (%) 1 - COVID-19 On Oxygen (%) 5 | 6.24 | 4.97 | 8.71 | 47 | *P<.*001 |
| Pair 5 | COVID-19 On Oxygen (%) 2 - COVID-19 On Oxygen (%) 3 | 6.61 | 3.89 | 23.3 | 187 | *P<.*001 |
| Pair 6 | COVID-19 On Oxygen (%) 3 - COVID-19 On Oxygen (%) 4 | 5.46 | 3.29 | 19.5 | 137 | *P<.*001 |
| Pair 7 | COVID-19 On Oxygen (%) 4 - COVID-19 On Oxygen (%) 5 | 0.40 | 2.62 | 1.73 | 126 | *P=.*0863 |
| Pair 1 | COVID-19 On Ventilator (%) 1 - COVID-19 On Ventilator (%) 2 | 2.07 | 1.47 | 15.7 | 124 | *P<.*001 |
| Pair 2 | COVID-19 On Ventilator (%) 1 - COVID-19 On Ventilator (%) 3 | 1.60 | 1.80 | 9.85 | 122 | *P<.*001 |
| Pair 3 | COVID-19 On Ventilator (%) 1 - COVID-19 On Ventilator (%) 4 | 2.91 | 0.63 | 32.8 | 50 | *P<.*001 |
| Pair 4 | COVID-19 On Ventilator (%) 1 - COVID-19 On Ventilator (%) 5 | 2.47 | 1.13 | 15.1 | 47 | *P<.*001 |
| Pair 5 | COVID-19 On Ventilator (%) 2 - COVID-19 On Ventilator (%) 3 | -1.21 | 1.52 | -10.9 | 187 | *P<.*001 |
| Pair 6 | COVID-19 On Ventilator (%) 3 - COVID-19 On Ventilator (%) 4 | 3.39 | 0.71 | 56.2 | 137 | *P<.*001 |
| Pair 7 | COVID-19 On Ventilator (%) 4 - COVID-19 On Ventilator (%) 5 | -0.10 | 1.84 | -0.62 | 126 | *P=.*54 |

Table S 10: Mean Paired Differences, Standard Deviation (Std.) Paired Differences, Standard Error of Mean (Std. Error Mean), 95% confidence interval (CI) if the Upper and Lower Difference, t-value, degrees of freedom (df) and P-value (Sig. (2-tailed) for the COVID-19 Hospitalised Cases in the Ages of 0 to 9, 10 to 19, 20 to 29, 30 to 39, 40 to 49, 50 to 59, 60 to 69, 70 to 79, 80 to 89 years in Pair 1 to Pair 7 **(Paired Samples T-Test**)

| **Paired Samples Test** | | | | | | |
| --- | --- | --- | --- | --- | --- | --- |
| **Sample T-test Pairing** | **Paired Variables** | **Paired Differences Mean** | **Paired Differences Std. Deviation** | **t** | **df** | **Sig. (2-tailed)** |
| Pair 1 | COVID-19 Hospitalised Admitted Age 10-19 years (%) 1 - COVID-19 Hospitalised Admitted Age 10-19 years (%) 2 | -0.37 | 0.29 | -13.3 | 108 | *P<.*001 |
| Pair 2 | COVID-19 Hospitalised Admitted Age 10-19 years (%) 1 - COVID-19 Hospitalised Admitted Age 10-19 years (%) 3 | -0.88 | 0.28 | -33.7 | 110 | *P<.*001 |
| Pair 3 | COVID-19 Hospitalised Admitted Age 10-19 years (%) 1 - COVID-19 Hospitalised Admitted Age 10-19 years (%) 4 | -1.88 | 0.18 | -76.1 | 50 | *P<.*001 |
| Pair 4 | COVID-19 Hospitalised Admitted Age 10-19 years (%) 1 - COVID-19 Hospitalised Admitted Age 10-19 years (%) 5 | -2.06 | 0.20 | -70.3 | 47 | *P<.*001 |
| Pair 5 | COVID-19 Hospitalised Admitted Age 10-19 years (%) 2 - COVID-19 Hospitalised Admitted Age 10-19 years (%) 3 | -0.40 | 0.31 | -17.2 | 179 | *P<.*001 |
| Pair 6 | COVID-19 Hospitalised Admitted Age 10-19 years (%) 3 - COVID-19 Hospitalised Admitted Age 10-19 years (%) 4 | -0.88 | 0.25 | -42.0 | 137 | *P<.*001 |
| Pair 7 | COVID-19 Hospitalised Admitted Age 10-19 years (%) 4 - COVID-19 Hospitalised Admitted Age 10-19 years (%) 5 | -0.36 | 0.15 | -27.8 | 126 | *P<.*001 |
| Pair 1 | COVID-19 Hospitalised Admitted Age 20-29 years (%) 1 - COVID-19 Hospitalised Admitted Age 20-29 years (%) 2 | 1.21 | 2.17 | 6.2 | 124 | *P<.*001 |
| Pair 2 | COVID-19 Hospitalised Admitted Age 20-29 years (%) 1 - COVID-19 Hospitalised Admitted Age 20-29 years (%) 3 | 0.76 | 2.29 | 3.7 | 122 | *P<.*001 |
| Pair 3 | COVID-19 Hospitalised Admitted Age 20-29 years (%) 1 - COVID-19 Hospitalised Admitted Age 20-29 years (%) 4 | 0.76 | 1.86 | 2.9 | 50 | *P=.*00511 |
| Pair 4 | COVID-19 Hospitalised Admitted Age 20-29 years (%) 1 - COVID-19 Hospitalised Admitted Age 20-29 years (%) 5 | 0.44 | 2.02 | 1.5 | 47 | *P=.*143 |
| Pair 5 | COVID-19 Hospitalised Admitted Age 20-29 years (%) 2 - COVID-19 Hospitalised Admitted Age 20-29 years (%) 3 | -0.35 | 3.10 | -1.6 | 187 | *P=.*120 |
| Pair 6 | COVID-19 Hospitalised Admitted Age 20-29 years (%) 3 - COVID-19 Hospitalised Admitted Age 20-29 years (%) 4 | -1.11 | 3.45 | -3.8 | 137 | *P<.*001 |
| Pair 7 | COVID-19 Hospitalised Admitted Age 20-29 years (%) 4 - COVID-19 Hospitalised Admitted Age 20-29 years (%) 5 | -0.41 | 0.33 | -13.9 | 126 | *P<.*001 |
| Pair 1 | COVID-19 Hospitalised Admitted Age 30-39 years (%) 1 - COVID-19 Hospitalised Admitted Age 30-39 years (%) 2 | 3.92 | 3.08 | 14.2 | 124 | *P<.*001 |
| Pair 2 | COVID-19 Hospitalised Admitted Age 30-39 years (%) 1 - COVID-19 Hospitalised Admitted Age 30-39 years (%) 3 | 3.97 | 3.36 | 13.1 | 122 | *P<.*001 |
| Pair 3 | COVID-19 Hospitalised Admitted Age 30-39 years (%) 1 - COVID-19 Hospitalised Admitted Age 30-39 years (%) 4 | 4.98 | 2.65 | 13.4 | 50 | *P<.*001 |
| Pair 4 | COVID-19 Hospitalised Admitted Age 30-39 years (%) 1 - COVID-19 Hospitalised Admitted Age 30-39 years (%) 5 | 4.69 | 2.86 | 11.4 | 47 | *P<.*001 |
| Pair 5 | COVID-19 Hospitalised Admitted Age 30-39 years (%) 2 - COVID-19 Hospitalised Admitted Age 30-39 years (%) 3 | 0.64 | 2.33 | 3.8 | 187 | *P<.*001 |
| Pair 6 | COVID-19 Hospitalised Admitted Age 30-39 years (%) 3 - COVID-19 Hospitalised Admitted Age 30-39 years (%) 4 | -0.88 | 0.94 | -10.9 | 137 | *P<.*001 |
| Pair 7 | COVID-19 Hospitalised Admitted Age 30-39 years (%) 4 - COVID-19 Hospitalised Admitted Age 30-39 years (%) 5 | -0.16 | 0.20 | -8.8 | 126 | *P<.*001 |
| Pair 1 | COVID-19 Hospitalised Admitted Age 40-49 years (%) 1 - COVID-19 Hospitalised Admitted Age 40-49 years (%) 2 | 2.84 | 2.38 | 10.2 | 72 | *P<.*001 |
| Pair 2 | COVID-19 Hospitalised Admitted Age 40-49 years (%) 1 - COVID-19 Hospitalised Admitted Age 40-49 years (%) 3 | 3.27 | 2.98 | 9.2 | 69 | *P<.*001 |
| Pair 3 | COVID-19 Hospitalised Admitted Age 40-49 years (%) 1 - COVID-19 Hospitalised Admitted Age 40-49 years (%) 4 | 6.70 | 3.48 | 8.8 | 20 | *P<.*001 |
| Pair 4 | COVID-19 Hospitalised Admitted Age 40-49 years (%) 1 - COVID-19 Hospitalised Admitted Age 40-49 years (%) 5 | 6.80 | 3.54 | 9.0 | 21 | *P<.*001 |
| Pair 5 | COVID-19 Hospitalised Admitted Age 40-49 years (%) 2 - COVID-19 Hospitalised Admitted Age 40-49 years (%) 3 | 0.60 | 1.70 | 4.7 | 179 | *P<.*001 |
| Pair 6 | COVID-19 Hospitalised Admitted Age 40-49 years (%) 3 - COVID-19 Hospitalised Admitted Age 40-49 years (%) 4 | 0.66 | 0.79 | 9.8 | 135 | *P<.*001 |
| Pair 7 | COVID-19 Hospitalised Admitted Age 40-49 years (%) 4 - COVID-19 Hospitalised Admitted Age 40-49 years (%) 5 | 0.53 | 0.28 | 21.7 | 126 | *P<.*001 |
| Pair 1 | COVID-19 Hospitalised Admitted Age 50-59 years (%) 1 - COVID-19 Hospitalised Admitted Age 50-59 years (%) 2 | 3.18 | 4.19 | 8.5 | 124 | *P<.*001 |
| Pair 2 | COVID-19 Hospitalised Admitted Age 50-59 years (%) 1 - COVID-19 Hospitalised Admitted Age 50-59 years (%) 3 | 3.40 | 4.45 | 8.4 | 121 | *P<.*001 |
| Pair 3 | COVID-19 Hospitalised Admitted Age 50-59 years (%) 1 - COVID-19 Hospitalised Admitted Age 50-59 years (%) 4 | 4.39 | 5.50 | 5.7 | 50 | *P<.*001 |
| Pair 4 | COVID-19 Hospitalised Admitted Age 50-59 years (%) 1 - COVID-19 Hospitalised Admitted Age 50-59 years (%) 5 | 4.69 | 5.59 | 5.8 | 47 | *P<.*001 |
| Pair 5 | COVID-19 Hospitalised Admitted Age 50-59 years (%) 2 - COVID-19 Hospitalised Admitted Age 50-59 years (%) 3 | 0.37 | 2.84 | 1.8 | 185 | *P=.*0801 |
| Pair 6 | COVID-19 Hospitalised Admitted Age 50-59 years (%) 3 - COVID-19 Hospitalised Admitted Age 50-59 years (%) 4 | 1.70 | 1.28 | 15.5 | 137 | *P<.*001 |
| Pair 7 | COVID-19 Hospitalised Admitted Age 50-59 years (%) 4 - COVID-19 Hospitalised Admitted Age 50-59 years (%) 5 | 0.90 | 0.47 | 21.4 | 126 | *P<.*001 |
| Pair 1 | COVID-19 Hospitalised Admitted Age 60-69 years (%) 1 - COVID-19 Hospitalised Admitted Age 60-69 years (%) 2 | -0.47 | 2.43 | -2.1 | 123 | *P=.*0342 |
| Pair 2 | COVID-19 Hospitalised Admitted Age 60-69 years (%) 1 - COVID-19 Hospitalised Admitted Age 60-69 years (%) 3 | 0.19 | 3.03 | 0.7 | 121 | *P=.*48 |
| Pair 3 | COVID-19 Hospitalised Admitted Age 60-69 years (%) 1 - COVID-19 Hospitalised Admitted Age 60-69 years (%) 4 | 0.92 | 3.54 | 1.9 | 50 | *P=.*0684 |
| Pair 4 | COVID-19 Hospitalised Admitted Age 60-69 years (%) 1 - COVID-19 Hospitalised Admitted Age 60-69 years (%) 5 | 1.11 | 3.56 | 2.2 | 47 | *P=.*0355 |
| Pair 5 | COVID-19 Hospitalised Admitted Age 60-69 years (%) 2 - COVID-19 Hospitalised Admitted Age 60-69 years (%) 3 | 0.07 | 2.25 | 0.4 | 186 | *P=.*676 |
| Pair 6 | COVID-19 Hospitalised Admitted Age 60-69 years (%) 3 - COVID-19 Hospitalised Admitted Age 60-69 years (%) 4 | 1.47 | 1.03 | 16.9 | 137 | *P<.*001 |
| Pair 7 | COVID-19 Hospitalised Admitted Age 60-69 years (%) 4 - COVID-19 Hospitalised Admitted Age 60-69 years (%) 5 | 0.55 | 0.22 | 28.1 | 126 | *P<.*001 |
| Pair 1 | COVID-19 Hospitalised Admitted Age 70-79 years (%) 1 - COVID-19 Hospitalised Admitted Age 70-79 years (%) 2 | -0.38 | 1.78 | -2.4 | 124 | *P=.*0176 |
| Pair 2 | COVID-19 Hospitalised Admitted Age 70-79 years (%) 1 - COVID-19 Hospitalised Admitted Age 70-79 years (%) 3 | -1.14 | 5.01 | -2.5 | 122 | *P=.*0132 |
| Pair 3 | COVID-19 Hospitalised Admitted Age 70-79 years (%) 1 - COVID-19 Hospitalised Admitted Age 70-79 years (%) 4 | -0.37 | 2.61 | -1.0 | 50 | *P=.*311 |
| Pair 4 | COVID-19 Hospitalised Admitted Age 70-79 years (%) 1 - COVID-19 Hospitalised Admitted Age 70-79 years (%) 5 | -0.41 | 2.65 | -1.1 | 47 | *P=.*288 |
| Pair 5 | COVID-19 Hospitalised Admitted Age 70-79 years (%) 2 - COVID-19 Hospitalised Admitted Age 70-79 years (%) 3 | -0.97 | 3.74 | -3.5 | 183 | *P<.*001 |
| Pair 6 | COVID-19 Hospitalised Admitted Age 70-79 years (%) 3 - COVID-19 Hospitalised Admitted Age 70-79 years (%) 4 | 0.49 | 0.74 | 7.7 | 137 | *P<.*001 |
| Pair 7 | COVID-19 Hospitalised Admitted Age 70-79 years (%) 4 - COVID-19 Hospitalised Admitted Age 70-79 years (%) 5 | 0.08 | 0.05 | 16.2 | 126 | *P<.*001 |
| Pair 1 | COVID-19 Hospitalised Admitted Age 80-89 years (%) 1 - COVID-19 Hospitalised Admitted Age 80-89 years (%) 2 | 0.49 | 1.40 | 3.9 | 123 | *P<.*001 |
| Pair 2 | COVID-19 Hospitalised Admitted Age 80-89 years (%) 1 - COVID-19 Hospitalised Admitted Age 80-89 years (%) 3 | 0.06 | 1.55 | 0.4 | 121 | *P=.*69 |
| Pair 3 | COVID-19 Hospitalised Admitted Age 80-89 years (%) 1 - COVID-19 Hospitalised Admitted Age 80-89 years (%) 4 | -0.42 | 1.98 | -1.5 | 50 | *P=.*14 |
| Pair 4 | COVID-19 Hospitalised Admitted Age 80-89 years (%) 1 - COVID-19 Hospitalised Admitted Age 80-89 years (%) 5 | -0.54 | 2.03 | -1.8 | 47 | *P=.*0707 |
| Pair 5 | COVID-19 Hospitalised Admitted Age 80-89 years (%) 2 - COVID-19 Hospitalised Admitted Age 80-89 years (%) 3 | -0.36 | 0.50 | -9.9 | 183 | *P<.*001 |
| Pair 6 | COVID-19 Hospitalised Admitted Age 80-89 years (%) 3 - COVID-19 Hospitalised Admitted Age 80-89 years (%) 4 | 0.04 | 0.35 | 1.5 | 137 | *P=.*15 |
| Pair 7 | COVID-19 Hospitalised Admitted Age 80-89 years (%) 4 - COVID-19 Hospitalised Admitted Age 80-89 years (%) 5 | -0.13 | 0.08 | -19.0 | 126 | *P<.*001 |

Table S 11: Mean Paired Differences, Standard Deviation (Std.) Paired Differences, Standard Error of Mean (Std. Error Mean), 95% confidence interval (CI) if the Upper and Lower Difference, t-value, degrees of freedom (df) and P-value (Sig. (2-tailed) for the COVID-19 Hospitalised Deaths in the Ages of 0 to 9, 10 to 19, 20 to 29, 30 to 39, 40 to 49, 50 to 59, 60 to 69, 70 to 79, 80 to 89 years in Pair 1 to Pair 7 **(Paired Samples T-Test**)

| **Paired Samples Test** | | | | | | |
| --- | --- | --- | --- | --- | --- | --- |
| **Sample T-test Pairing** | **Paired Variables** | **Paired Differences Mean** | **Paired Differences Std. Deviation** | **t** | **df** | **Sig. (2-tailed)** |
| Pair 1 | COVID-19 Hospitalised Deaths Age 10-19 years (%) 1 - COVID-19 Hospitalised Deaths Age 10-19 years (%) 2 | -0.005 | 0.151 | -0.37 | 124 | *P=.*71 |
| Pair 2 | COVID-19 Hospitalised Deaths Age 10-19 years (%) 1 - COVID-19 Hospitalised Deaths Age 10-19 years (%) 3 | -0.029 | 0.164 | -1.96 | 122 | *P=.*0519 |
| Pair 3 | COVID-19 Hospitalised Deaths Age 10-19 years (%) 1 - COVID-19 Hospitalised Deaths Age 10-19 years (%) 4 | -0.986 | 0.815 | -8.64 | 50 | *P<.*001 |
| Pair 4 | COVID-19 Hospitalised Deaths Age 10-19 years (%) 1 - COVID-19 Hospitalised Deaths Age 10-19 years (%) 5 | -0.761 | 0.083 | -63.55 | 47 | *P<.*001 |
| Pair 5 | COVID-19 Hospitalised Deaths Age 10-19 years (%) 2 - COVID-19 Hospitalised Deaths Age 10-19 years (%) 3 | 0.014 | 0.084 | 2.28 | 181 | *P=.*0238 |
| Pair 6 | COVID-19 Hospitalised Deaths Age 10-19 years (%) 3 - COVID-19 Hospitalised Deaths Age 10-19 years (%) 4 | -0.771 | 0.580 | -15.40 | 133 | *P<.*001 |
| Pair 7 | COVID-19 Hospitalised Deaths Age 10-19 years (%) 4 - COVID-19 Hospitalised Deaths Age 10-19 years (%) 5 | 0.180 | 0.771 | 2.60 | 122 | *P=*0.0106 |
| Pair 1 | COVID-19 Hospitalised Deaths Age 20-29 years (%) 1 - COVID-19 Hospitalised Deaths Age 20-29 years (%) 2 | 0.024 | 0.661 | 0.40 | 124 | *P=.*69 |
| Pair 2 | COVID-19 Hospitalised Deaths Age 20-29 years (%) 1 - COVID-19 Hospitalised Deaths Age 20-29 years (%) 3 | 0.221 | 0.619 | 3.96 | 122 | *P<.*001 |
| Pair 3 | COVID-19 Hospitalised Deaths Age 20-29 years (%) 1 - COVID-19 Hospitalised Deaths Age 20-29 years (%) 4 | -3.647 | 3.347 | -7.78 | 50 | *P<.*001 |
| Pair 4 | COVID-19 Hospitalised Deaths Age 20-29 years (%) 1 - COVID-19 Hospitalised Deaths Age 20-29 years (%) 5 | -2.773 | 0.253 | -75.88 | 47 | *P<.*001 |
| Pair 5 | COVID-19 Hospitalised Deaths Age 20-29 years (%) 2 - COVID-19 Hospitalised Deaths Age 20-29 years (%) 3 | 0.321 | 0.500 | 8.69 | 182 | *P<.*001 |
| Pair 6 | COVID-19 Hospitalised Deaths Age 20-29 years (%) 3 - COVID-19 Hospitalised Deaths Age 20-29 years (%) 4 | -3.515 | 2.159 | -18.98 | 135 | *P<.*001 |
| Pair 7 | COVID-19 Hospitalised Deaths Age 20-29 years (%) 4 - COVID-19 Hospitalised Deaths Age 20-29 years (%) 5 | -0.116 | 4.781 | -0.27 | 126 | *P=.*78 |
| Pair 1 | COVID-19 Hospitalised Deaths Age 30-39 years (%) 1 - COVID-19 Hospitalised Deaths Age 30-39 years (%) 2 | 1.264 | 1.994 | 7.09 | 124 | *P<.*001 |
| Pair 2 | COVID-19 Hospitalised Deaths Age 30-39 years (%) 1 - COVID-19 Hospitalised Deaths Age 30-39 years (%) 3 | 1.020 | 2.188 | 5.17 | 122 | *P<.*001 |
| Pair 3 | COVID-19 Hospitalised Deaths Age 30-39 years (%) 1 - COVID-19 Hospitalised Deaths Age 30-39 years (%) 4 | -6.269 | 8.114 | -5.52 | 50 | *P<.*001 |
| Pair 4 | COVID-19 Hospitalised Deaths Age 30-39 years (%) 1 - COVID-19 Hospitalised Deaths Age 30-39 years (%) 5 | -4.803 | 0.778 | -42.78 | 47 | *P<.*001 |
| Pair 5 | COVID-19 Hospitalised Deaths Age 30-39 years (%) 2 - COVID-19 Hospitalised Deaths Age 30-39 years (%) 3 | -0.518 | 1.416 | -4.96 | 183 | *P<.*001 |
| Pair 6 | COVID-19 Hospitalised Deaths Age 30-39 years (%) 3 - COVID-19 Hospitalised Deaths Age 30-39 years (%) 4 | -6.941 | 6.325 | -12.89 | 137 | *P<.*001 |
| Pair 7 | COVID-19 Hospitalised Deaths Age 30-39 years (%) 4 - COVID-19 Hospitalised Deaths Age 30-39 years (%) 5 | 1.512 | 6.982 | 2.44 | 126 | *P=.*0161 |
| Pair 1 | COVID-19 Hospitalised Deaths Age 40-49 years (%) 1 - COVID-19 Hospitalised Deaths Age 40-49 years (%) 2 | 3.961 | 4.744 | 9.33 | 124 | *P<.*001 |
| Pair 2 | COVID-19 Hospitalised Deaths Age 40-49 years (%) 1 - COVID-19 Hospitalised Deaths Age 40-49 years (%) 3 | 3.431 | 4.745 | 8.02 | 122 | *P<.*001 |
| Pair 3 | COVID-19 Hospitalised Deaths Age 40-49 years (%) 1 - COVID-19 Hospitalised Deaths Age 40-49 years (%) 4 | -1.242 | 9.218 | -0.96 | 50 | *P=.*341 |
| Pair 4 | COVID-19 Hospitalised Deaths Age 40-49 years (%) 1 - COVID-19 Hospitalised Deaths Age 40-49 years (%) 5 | 0.188 | 1.286 | 1.01 | 47 | *P=.*316 |
| Pair 5 | COVID-19 Hospitalised Deaths Age 40-49 years (%) 2 - COVID-19 Hospitalised Deaths Age 40-49 years (%) 3 | -0.769 | 1.522 | -6.85 | 183 | *P<.*001 |
| Pair 6 | COVID-19 Hospitalised Deaths Age 40-49 years (%) 3 - COVID-19 Hospitalised Deaths Age 40-49 years (%) 4 | -5.822 | 7.186 | -9.52 | 137 | *P<.*001 |
| Pair 7 | COVID-19 Hospitalised Deaths Age 40-49 years (%) 4 - COVID-19 Hospitalised Deaths Age 40-49 years (%) 5 | 1.245 | 7.611 | 1.84 | 125 | *P=.*0686 |
| Pair 1 | COVID-19 Hospitalised Deaths Age 50-59 years (%) 1 - COVID-19 Hospitalised Deaths Age 50-59 years (%) 2 | 6.119 | 3.222 | 16.67 | 76 | *P<.*001 |
| Pair 2 | COVID-19 Hospitalised Deaths Age 50-59 years (%) 1 - COVID-19 Hospitalised Deaths Age 50-59 years (%) 3 | 3.517 | 2.765 | 11.09 | 75 | *P<.*001 |
| Pair 3 | COVID-19 Hospitalised Deaths Age 50-59 years (%) 1 - COVID-19 Hospitalised Deaths Age 50-59 years (%) 4 | 9.441 | 11.210 | 6.01 | 50 | *P<.*001 |
| Pair 4 | COVID-19 Hospitalised Deaths Age 50-59 years (%) 1 - COVID-19 Hospitalised Deaths Age 50-59 years (%) 5 | 11.074 | 2.951 | 26.00 | 47 | *P<.*001 |
| Pair 5 | COVID-19 Hospitalised Deaths Age 50-59 years (%) 2 - COVID-19 Hospitalised Deaths Age 50-59 years (%) 3 | -3.464 | 1.651 | -28.15 | 179 | *P<.*001 |
| Pair 6 | COVID-19 Hospitalised Deaths Age 50-59 years (%) 3 - COVID-19 Hospitalised Deaths Age 50-59 years (%) 4 | 3.669 | 7.184 | 6.00 | 137 | *P<.*001 |
| Pair 7 | COVID-19 Hospitalised Deaths Age 50-59 years (%) 4 - COVID-19 Hospitalised Deaths Age 50-59 years (%) 5 | -0.124 | 9.064 | -0.15 | 126 | *P=.*88 |
| Pair 1 | COVID-19 Hospitalised Deaths Age 60-69 years (%) 1 - COVID-19 Hospitalised Deaths Age 60-69 years (%) 2 | 3.629 | 3.993 | 10.12 | 123 | *P<.*001 |
| Pair 2 | COVID-19 Hospitalised Deaths Age 60-69 years (%) 1 - COVID-19 Hospitalised Deaths Age 60-69 years (%) 3 | 4.293 | 4.209 | 11.27 | 121 | *P<.*001 |
| Pair 3 | COVID-19 Hospitalised Deaths Age 60-69 years (%) 1 - COVID-19 Hospitalised Deaths Age 60-69 years (%) 4 | 7.949 | 2.233 | 24.92 | 48 | *P<.*001 |
| Pair 4 | COVID-19 Hospitalised Deaths Age 60-69 years (%) 1 - COVID-19 Hospitalised Deaths Age 60-69 years (%) 5 | 10.295 | 2.434 | 29.31 | 47 | *P<.*001 |
| Pair 5 | COVID-19 Hospitalised Deaths Age 60-69 years (%) 2 - COVID-19 Hospitalised Deaths Age 60-69 years (%) 3 | -3.101 | 6.266 | -6.69 | 182 | *P<.*001 |
| Pair 6 | COVID-19 Hospitalised Deaths Age 60-69 years (%) 3 - COVID-19 Hospitalised Deaths Age 60-69 years (%) 4 | 9.487 | 3.574 | 30.61 | 132 | *P<.*001 |
| Pair 7 | COVID-19 Hospitalised Deaths Age 60-69 years (%) 4 - COVID-19 Hospitalised Deaths Age 60-69 years (%) 5 | 2.767 | 4.237 | 7.24 | 122 | *P<.*001 |
| Pair 1 | COVID-19 Hospitalised Deaths Age 70-79 years (%) 1 - COVID-19 Hospitalised Deaths Age 70-79 years (%) 2 | 1.926 | 2.821 | 7.60 | 123 | *P<.*001 |
| Pair 2 | COVID-19 Hospitalised Deaths Age 70-79 years (%) 1 - COVID-19 Hospitalised Deaths Age 70-79 years (%) 3 | -1.461 | 3.662 | -4.41 | 121 | *P<.*001 |
| Pair 3 | COVID-19 Hospitalised Deaths Age 70-79 years (%) 1 - COVID-19 Hospitalised Deaths Age 70-79 years (%) 4 | -6.089 | 16.005 | -2.72 | 50 | *P=*0.00902 |
| Pair 4 | COVID-19 Hospitalised Deaths Age 70-79 years (%) 1 - COVID-19 Hospitalised Deaths Age 70-79 years (%) 5 | -2.510 | 1.563 | -11.12 | 47 | *P<.*001 |
| Pair 5 | COVID-19 Hospitalised Deaths Age 70-79 years (%) 2 - COVID-19 Hospitalised Deaths Age 70-79 years (%) 3 | -6.204 | 4.904 | -17.16 | 183 | *P<.*001 |
| Pair 6 | COVID-19 Hospitalised Deaths Age 70-79 years (%) 3 - COVID-19 Hospitalised Deaths Age 70-79 years (%) 4 | 3.052 | 10.658 | 3.36 | 137 | *P=.*000996 |
| Pair 7 | COVID-19 Hospitalised Deaths Age 70-79 years (%) 4 - COVID-19 Hospitalised Deaths Age 70-79 years (%) 5 | 1.128 | 12.817 | 0.99 | 126 | *P=.*32 |
| Pair 1 | COVID-19 Hospitalised Deaths Age 80-89 years (%) 1 - COVID-19 Hospitalised Deaths Age 80-89 years (%) 2 | 3.910 | 2.912 | 14.95 | 123 | *P<.*001 |
| Pair 2 | COVID-19 Hospitalised Deaths Age 80-89 years (%) 1 - COVID-19 Hospitalised Deaths Age 80-89 years (%) 3 | -0.690 | 3.752 | -2.03 | 121 | *P=.*0445 |
| Pair 3 | COVID-19 Hospitalised Deaths Age 80-89 years (%) 1 - COVID-19 Hospitalised Deaths Age 80-89 years (%) 4 | -10.272 | 14.178 | -5.17 | 50 | *P<.*001 |
| Pair 4 | COVID-19 Hospitalised Deaths Age 80-89 years (%) 1 - COVID-19 Hospitalised Deaths Age 80-89 years (%) 5 | -10.023 | 2.226 | -31.19 | 47 | *P<.*001 |
| Pair 5 | COVID-19 Hospitalised Deaths Age 80-89 years (%) 2 - COVID-19 Hospitalised Deaths Age 80-89 years (%) 3 | -5.702 | 2.397 | -32.26 | 183 | *P<.*001 |
| Pair 6 | COVID-19 Hospitalised Deaths Age 80-89 years (%) 3 - COVID-19 Hospitalised Deaths Age 80-89 years (%) 4 | -3.187 | 8.730 | -4.26 | 135 | *P<.*001 |
| Pair 7 | COVID-19 Hospitalised Deaths Age 80-89 years (%) 4 - COVID-19 Hospitalised Deaths Age 80-89 years (%) 5 | -1.220 | 14.777 | -0.92 | 124 | *P=.*36 |

Table S 12: Mean Paired Differences, Standard Deviation (Std.) Paired Differences, Standard Error of Mean (Std. Error Mean), 95% confidence interval (CI) if the Upper and Lower Difference, t-value, degrees of freedom (df) and P-value (Sig. (2-tailed) for the COVID-19 Hospital Case Fatality Rate (COVID-19 CFR), Hospital Discharge Rate (COVID-19 DR), Natural Deaths, Excess Natural Deaths, Weekly Reported COVID-19 Deaths and the Weekly Unreported Excess Deaths (Natural) to COVID-19 Death Ratio (ECDR) in Pair 1 to Pair 7. **(Paired Samples T-Test**)

| **Paired Samples Test** | | | | | | |
| --- | --- | --- | --- | --- | --- | --- |
| Sample T-test Pairing | Paired Variables | Paired Differences Mean | Paired Differences Std. Deviation | t | df | Sig. (2-tailed) |
| Pair 1 | CFR1 - CFR2 | -0.354 | 1.601 | -2.38 | 115 | *P=.*0189 |
| Pair 2 | CFR1 - CFR3 | 0.424 | 1.662 | 2.75 | 115 | *P=.*00702 |
| Pair 3 | CFR1 - CFR4 | 1.123 | 0.770 | 9.56 | 42 | *P<.*001 |
| Pair 4 | CFR1 - CFR5 | 0.952 | 0.813 | 6.93 | 34 | *P<.*001 |
| Pair 5 | CFR2 - CFR3 | 0.512 | 1.897 | 3.60 | 177 | *P<.*001 |
| Pair 6 | CFR3 - CFR4 | 0.533 | 7.702 | 0.79 | 128 | *P=.*43 |
| Pair 7 | CFR4 - CFR5 | 0.823 | 8.270 | 1.04 | 109 | *P=.*30 |
| Pair 1 | DR1 - DR2 | 0.817 | 7.883 | 1.12 | 115 | *P=.*27 |
| Pair 2 | DR1 - DR3 | 2.368 | 5.680 | 4.53 | 117 | *P<.*001 |
| Pair 3 | DR1 - DR4 | -0.286 | 6.127 | -0.32 | 45 | *P=.*75 |
| Pair 4 | DR1 - DR5 | -0.732 | 4.966 | -0.87 | 34 | *P=.*39 |
| Pair 5 | DR2 - DR3 | 1.768 | 9.125 | 2.60 | 179 | *P=.*0101 |
| Pair 6 | DR3 - DR4 | -2.372 | 8.010 | -3.45 | 135 | *P=.*000741 |
| Pair 7 | DR4 - DR5 | -0.955 | 12.476 | -0.83 | 117 | *P=.*41 |
| Pair 1 | ECDR1 - ECDR2 | -0.108 | 2.335 | -0.23 | 23 | *P=.*82 |
| Pair 2 | ECDR1 - ECDR3 | -0.214 | 2.914 | -0.36 | 23 | *P=.*72 |
| Pair 3 | ECDR1 - ECDR4 | -0.530 | 5.005 | -0.41 | 14 | *P=.*69 |
| Pair 4 | ECDR1 - ECDR5 | -7.393 | 5.405 | -4.93 | 12 | *P<.*001 |
| Pair 5 | ECDR2 - ECDR3 | -0.797 | 1.982 | -2.24 | 30 | *P=.*0328 |
| Pair 6 | ECDR3 - ECDR4 | -2.565 | 5.837 | -1.97 | 19 | *P=.*0641 |
| Pair 7 | ECDR4 - ECDR5 | -8.450 | 7.012 | -5.11 | 17 | *P<.*001 |
| Pair 1 | Weekly COVID-19 Deaths 1 - Weekly COVID-19 Deaths 2 | -803 | 980 | -4.42 | 28 | *P<.*001 |
| Pair 2 | Weekly COVID-19 Deaths 1 - Weekly COVID-19 Deaths 3 | -673 | 860 | -4.21 | 28 | *P<.*001 |
| Pair 3 | Weekly COVID-19 Deaths 1 - Weekly COVID-19 Deaths 4 | -16 | 888 | -0.08 | 19 | *P=.*94 |
| Pair 4 | Weekly COVID-19 Deaths 1 - Weekly COVID-19 Deaths 5 | 411 | 653 | 2.82 | 19 | *P=.*0110 |
| Pair 5 | Weekly COVID-19 Deaths 2 - Weekly COVID-19 Deaths 3 | 128.129 | 733.728 | 0.97 | 30 | *P=.*34 |
| Pair 6 | Weekly COVID-19 Deaths 3 - Weekly COVID-19 Deaths 4 | 1061.650 | 931.593 | 5.10 | 19 | *P<.*001 |
| Pair 7 | Weekly COVID-19 Deaths 4 - Weekly COVID-19 Deaths 5 | 427.300 | 410.538 | 4.65 | 19 | *P<.*001 |
| Pair 1 | SOUTH AFRICA Excess Deaths1 - SOUTH AFRICA Excess Deaths2 | -2371.750 | 3521.009 | -3.30 | 23 | *P=.*00313 |
| Pair 2 | SOUTH AFRICA Excess Deaths1 - SOUTH AFRICA Excess Deaths3 | -2045.750 | 3390.661 | -2.96 | 23 | *P=.*00709 |
| Pair 3 | SOUTH AFRICA Excess Deaths1 - SOUTH AFRICA Excess Deaths4 | 1741.764 | 2948.972 | 2.29 | 14 | *P=.*0382 |
| Pair 4 | SOUTH AFRICA Excess Deaths1 - SOUTH AFRICA Excess Deaths5 | 1835.844 | 3075.599 | 2.31 | 14 | *P=.*0365 |
| Pair 5 | SOUTH AFRICA Excess Deaths2 - SOUTH AFRICA Excess Deaths3 | 60.097 | 3404.805 | 0.10 | 30 | *P=.*92 |
| Pair 6 | SOUTH AFRICA Excess Deaths3 - SOUTH AFRICA Excess Deaths4 | 3538.723 | 3354.088 | 4.72 | 19 | *P<.*001 |
| Pair 7 | SOUTH AFRICA Excess Deaths4 - SOUTH AFRICA Excess Deaths5 | 415.395 | 790.786 | 2.35 | 19 | *P=.*0298 |
