# Supplementary figures and images for: The Impact of SARS-CoV-2 Lineages (Variants) and COVID-19 Vaccination on the COVID-19 Epidemic in South Africa: Regression Study
Source: JMIRx Med. 2023 Jul 3;4:e34598. doi: 10.2196/34598 (PMC10337479; doi:10.2196/34598)

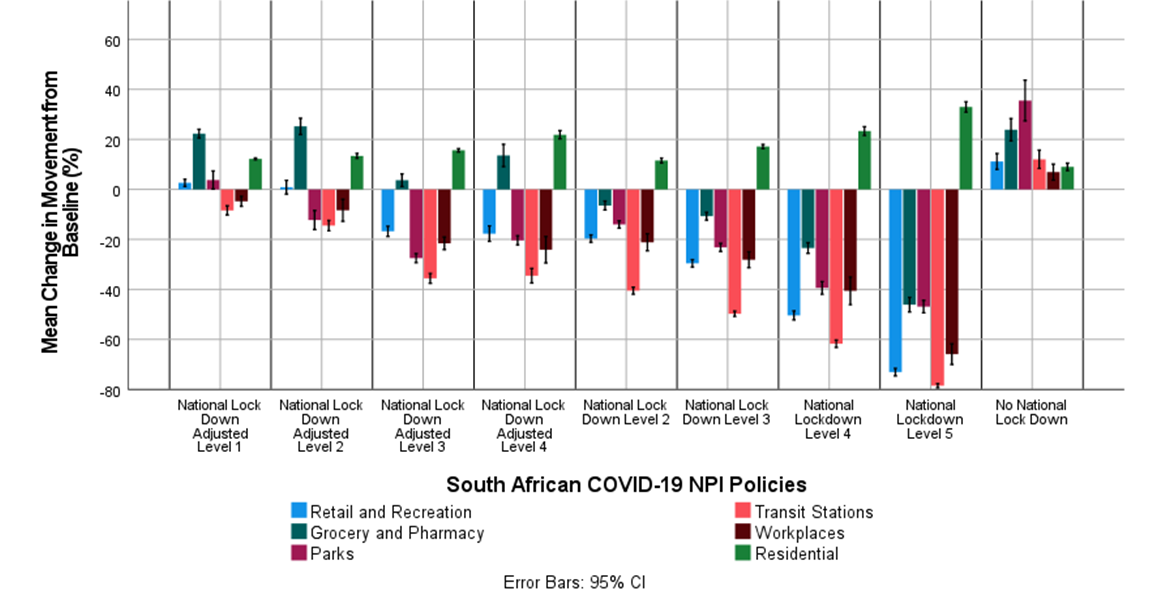

Supplement: Multimedia Appendix 2 [file xmed_v4i1e34598_app2.png]

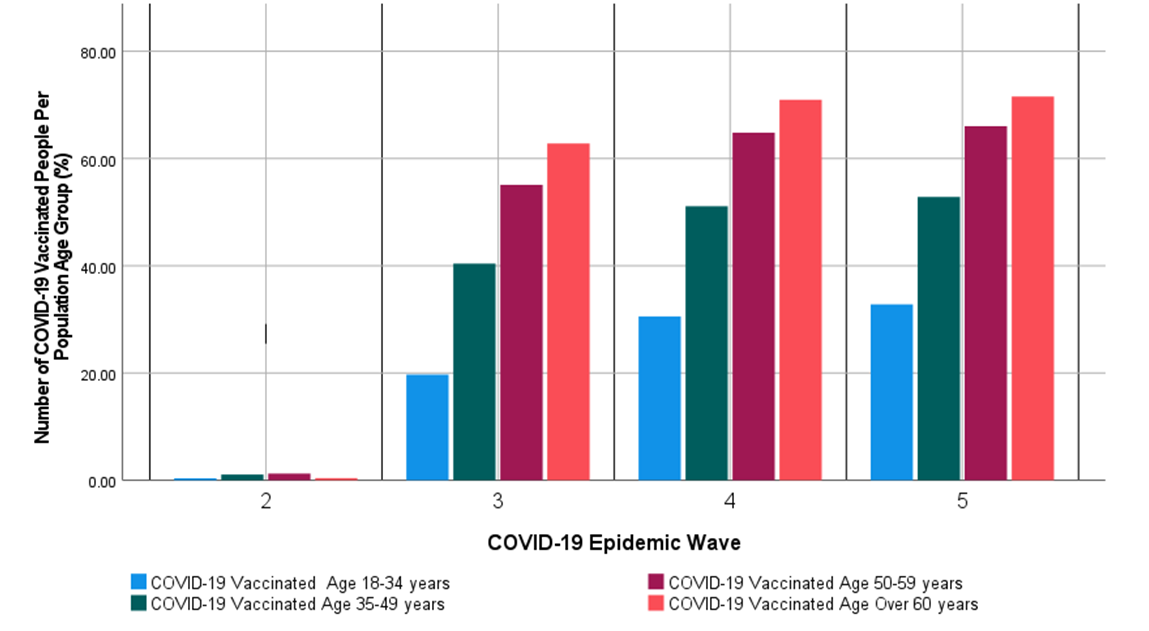

Supplement: Multimedia Appendix 3 [file xmed_v4i1e34598_app3.png]

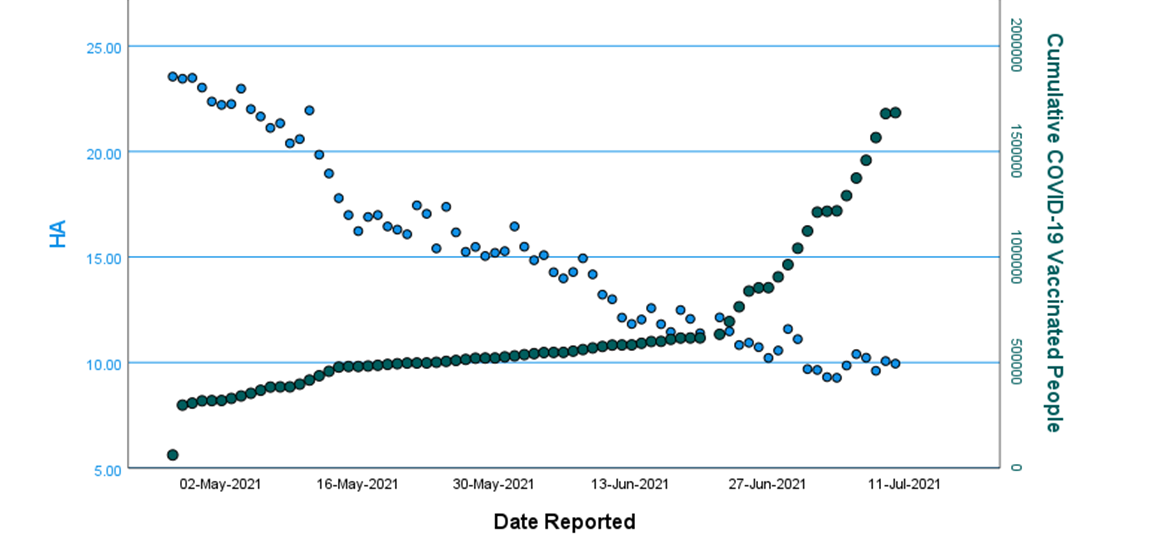

Supplement: Multimedia Appendix 4 [file xmed_v4i1e34598_app4.png]

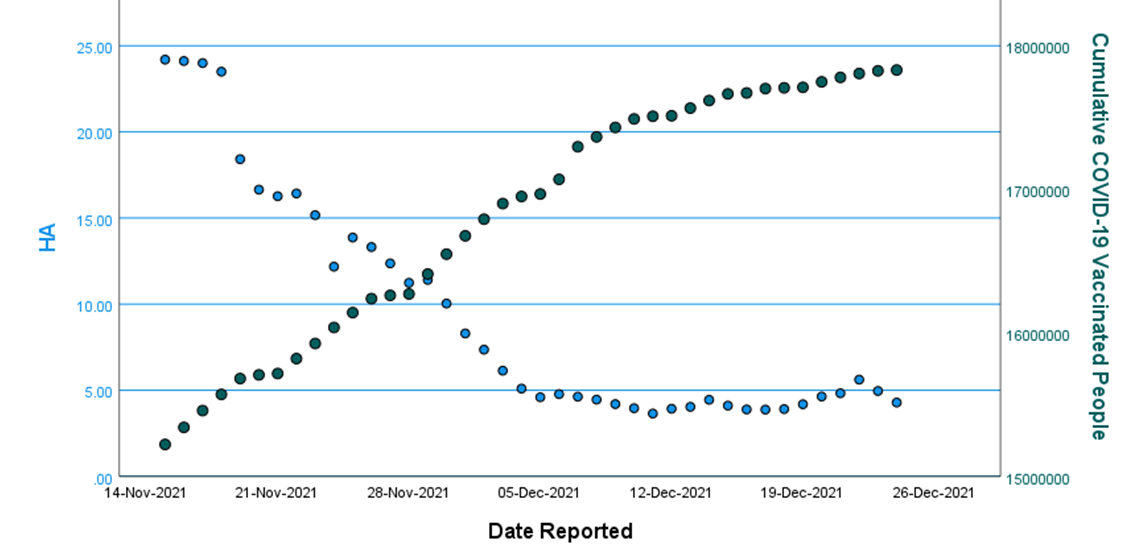

Supplement: Multimedia Appendix 5 [file xmed_v4i1e34598_app5.png]

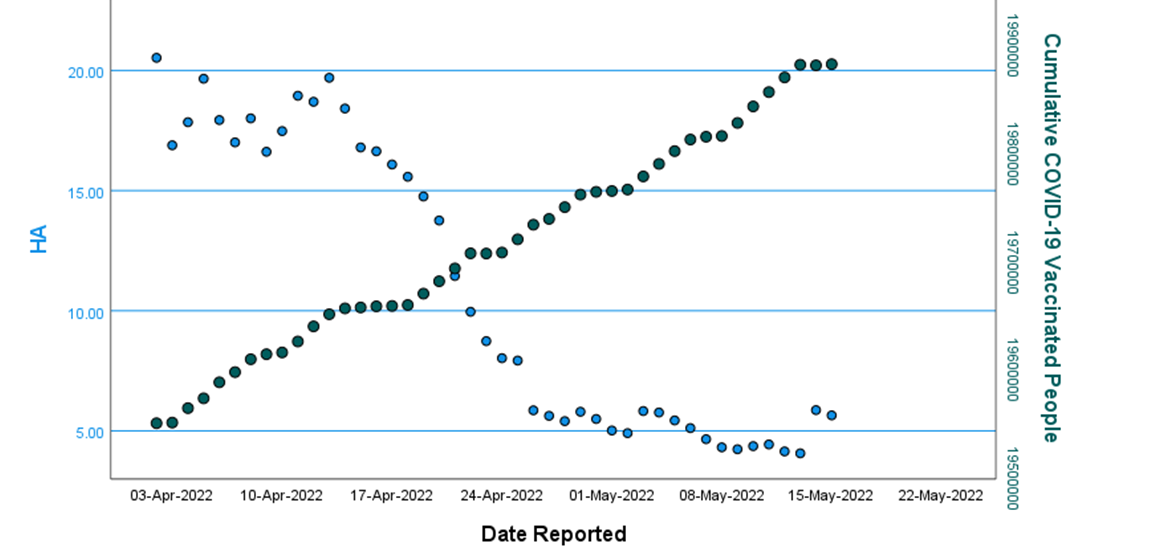

Supplement: Multimedia Appendix 6 [file xmed_v4i1e34598_app6.png]
